# Association of nighttime fasting duration, breakfast time and dinner time with healthy aging in Chinese older adults: a cross‑sectional study

**DOI:** 10.1186/s12937-026-01317-7

**Published:** 2026-03-23

**Authors:** Jiaming Fang, Liuhong Tian, Lingfang Wang, Jinghai Li, Shulei Chen, Xiaodan Kuang, Weihong Lin, Hongying Shi

**Affiliations:** 1 Department of Epidemiology and Health Statistics, School of Public Health, Wenzhou Medical University, Wenzhou, Zhejiang 325035 China; 2 Healthcare Center, The First Affiliated Hospital of Wenzhou Medical University, Wenzhou, Zhejiang 325000 China; 3 Wenzhou Key Laboratory of Intelligent Prevention and Active Health for Aging-Related Chronic Diseases, The First Affiliated Hospital of Wenzhou Medical University, Wenzhou, Zhejiang 325000 China

**Keywords:** Chrononutrition, Nighttime fasting duration, Meal timing, Healthy aging, Older adults

## Abstract

**Background:**

Proper eating/fasting cycle can regulate circadian rhythms, thereby influencing metabolic regulation, oxidative stress, and cognitive function. However, its association with overall health remains understudied. This study aimed to explore the relationship between nighttime fasting duration (NFD), breakfast time, dinner time and healthy aging among Chinese older adults.

**Methods:**

A cross-sectional study was conducted between September 2021 and December 2023, involving 947 older adults aged 60 years and above from various socioeconomic regions in China. Data on NFD and meal timing were obtained through a validated dietary behavior questionnaire. Healthy aging was defined as free of major chronic diseases, no limitation in physical function, no cognitive impairment, and no depressive symptoms. Logistic regression models were used to analyze the relationship between the NFD, breakfast time and dinner time, and healthy aging.

**Results:**

A total of 901 participants were included, with a median age of 69.00 years (IQR: 64.50, 74.00) and 56.60% being female. Of these, 29.86% met the criteria for healthy aging. An inverted U-shaped relationship was observed between NFD and healthy aging (*P*_nonlinear_ = 0.001). Compared to participants with 13 h ≤ NFD<14 h, those with NFD<12 h, 14 h ≤ NFD<15 h, and NFD≥15 h had lower odds of healthy aging, with adjusted ORs of 0.46 (95% CI: 0.23, 0.93), 0.62 (95% CI: 0.41, 0.95), and 0.34 (95% CI: 0.15, 0.77), respectively. A similar inverted U-shaped association was found for both breakfast time (*P*_nonlinear_ = 0.028) and dinner time (*P*_nonlinear_ = 0.007). Compared with participants who had breakfast 6:00–7:00, those who had breakfast before 6:00 or after 7:00 had lower odds of healthy aging, with adjusted ORs of 0.68 (95% CI: 0.47, 0.98), 0.78 (95% CI: 0.44, 1.40), respectively. Compared with participants who had dinner 17:00–18:00, those who had dinner before 17:00 or after 18:00 had lower odds of healthy aging, with adjusted ORs of 0.48 (95% CI: 0.23, 1.00), 0.49 (95% CI: 0.29, 0.82), respectively.

**Conclusions:**

Optimal circadian-related eating patterns, specifically a nighttime fasting duration of 13h to < 14 h, breakfast between 6:00 and 7:00, and dinner between 17:00 and 18:00, were associated with the highest odds of healthy aging among Chinese older adults.

**Supplementary Information:**

The online version contains supplementary material available at 10.1186/s12937-026-01317-7.

## Introduction

With the increase of life expectancy, the proportion of older adults in the population is steadily growing. By 2050, it is projected that the global population aged 60 years and older will rise from 1 billion in 2020 to approximately 2.1 billion [1]. Statistics from the World Health Organization show that as of 2021, the global life expectancy is 71.4 years, while the healthy life expectancy is only 61.9 years [2]. This indicates that a growing number of adverse health events, such as cardiovascular diseases, physical limitations, and cognitive impairments, are plaguing older adults [3]. Therefore, how to maintain physical, mental, and cognitive health and prevent chronic diseases during the aging process (i.e., healthy aging) has become a focus of public attention. China, as the most populous country, has the largest number of older adults [4]. However, the prevalence of healthy aging among Chinese older adults was less than 20% [5], underscoring the importance of identifying modifiable determinants of healthy aging.

Previous research has explored the association between dietary quality [6] and quantity [7] with healthy aging; however, no studies have explored the association between meal timing and healthy aging. Meal timing, second only to light exposure, is a significant time cue for circadian rhythms [8]. It influences human health by regulating peripheral circadian clocks [9]. Most studies suggest that time-restricted eating (TRE), which involves consuming all daily nutrition within 4 to 12 h without altering diet quality or caloric intake [10], and extending nighttime fasting duration (NFD), can improve health by reshaping circadian rhythms [11]. However, some studies reported that prolonged NFD was associated with poorer physical [12] and cognitive functions [13] in older adults, as well as biomarkers reflecting renal function, inflammation, and nutritional status [14]. Another study reported a U-shaped association between NFD and the risks of cardiovascular diseases and all-cause mortality, with the lowest risk observed at 10–11 h of NFD [15].

The inconsistency in prior studies may be due to the failure to consider the start and end points of the NFD. Recent evidence found that only among individuals consuming breakfast before 8:00, fasting for over 13 h was associated with a lower risk of type 2 diabetes [16], suggesting that meal timing might modify the health effects of NFD. However, studies investigating meal timing and health outcomes have also yielded conflicting results. Several cohort studies reported that delaying the first or last meal was associated with higher risks of cardiovascular diseases, type 2 diabetes, and selected cancers [16–18]. In contrast, other research found no association between first meal time and type 2 diabetes [19], while one cohort study of older adults aged 65 years observed that delaying the breakfast (> 9:00) was associated with a lower risk of type 2 diabetes [20]. These discrepancies may be related to the focus of prior studies on single health outcomes, and with limited attention given to older adults. As age increases, circadian rhythms gradually weaken [21], which may also lead to differences in results. Moreover, adults across different countries exhibit different meal timing patterns. For example, the average time for the first and last meals of American adults is 8:04 and 20:11 [22], for Austrians it is 7:30 and 18:30 [23], and for Japanese it is 7:31 and 19:57 [24]. This may also contribute to the inconsistencies observed in previous findings. Therefore, it is necessary to investigate the comprehensive health effects of NFD and meal timing in more diverse populations.

In light of this, this study aims to explore the relationship between NFD, breakfast time, dinner time, and healthy aging and its four dimensions among Chinese older adults; and to analyze potential interactions between NFD and meal timing, with the expectation of providing new insights into the potential role of eating behaviors in healthy aging.

## Methods

### Study design

This cross-sectional study was conducted between September 2021 and December 2023 among community-dwelling older adults in China. Participants were eligible if they: (1) were aged 60 years or older; (2) had no eating occasions reported from dinner to the next day’s breakfast; and (3) had no self-reported hearing or vision impairments. To ensure sample representativeness, older adults were recruited from 13 regions across Zhejiang, Anhui, Guizhou, Jiangxi, and Henan provinces, representing areas with varying economic conditions. GPower3.1 software was applied to estimate the required sample size for this study. Based on previous studies, the rate of healthy aging in China is approximately 13.7%−19.7% [5, 25, 26], with α set at 0.05 (two-sided), an Odds Ratio of 0.75–0.78 [17, 19, 27], and a statistical power of 0.80, the required sample size for this study was 419–740 individuals. Taking into account the non-response rate (15%), the final sample size was determined to be 482–851 individuals.

To minimize communication barriers, investigators proficient in local dialects were prioritized and received systematic training. Data were collected through face-to-face interviews, verifiers can review the data at any time and promptly contact respondents if they identify missing items or logical errors, ensuring the data’s authenticity and completeness. The research protocol has been reviewed and approved by the Ethics Committee of Wenzhou Medical University (Approval Number: 2020-060). All participants signed the informed consent form.

**Fig. 1:**
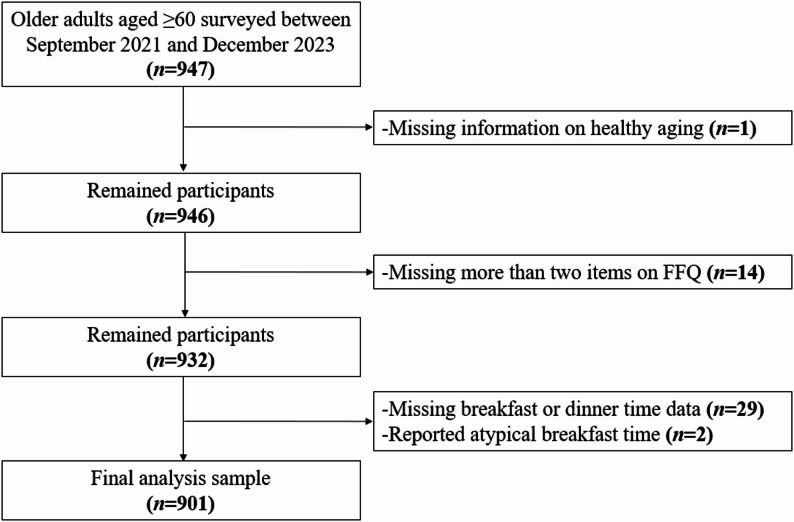
The flow chart for selecting study participants

Among the 947 older adults surveyed, we excluded those with missing data on healthy aging (*n* = 1), incomplete responses to the food frequency questionnaire (i.e., more than two missing items; *n* = 14), missing breakfast or dinner time data (*n* = 29), or atypical breakfast time (e.g., before 4:00 or after 9:00; *n* = 2). The final analysis included 901 participants (Fig. 1). No significant differences were observed between the included and excluded individuals in terms of age, sex, smoking status, drinking status, BMI, shift work experience, or sleep duration (Table S1).

### Evaluation of nighttime fasting duration, breakfast time, and dinner time

Data on meal timing were obtained using a validated dietary behavior questionnaire. Participants were asked: “How many meals do you usually eat in a day?” and “At what time do you usually have breakfast, lunch, and dinner?” Nighttime fasting duration was defined as the time interval between the last meal (dinner) and the first meal of the next day (breakfast) and was calculated using the formula: NFD = 24 − dinner time + breakfast time. Since TRE usually requires eating within 12 h [10], and based on the restricted cubic spline (RCS) analyses of NFD in relation to healthy aging as well as the data distribution, participants were categorized into five groups based on NFD: <12 h, 12 h ≤ NFD<13 h, 13 h ≤ NFD<14 h, 14 h ≤ NFD<15 h, and ≥ 15 h. Based on the RCS analyses of breakfast timing and dinner timing in relation to healthy aging, breakfast timing was grouped as < 6:00, 6:00–7:00, and > 7:00, while dinner timing was classified as < 17:00, 17:00–18:00, and > 18:00.

To validate the effectiveness of the dietary behavior questionnaire, six months after the first survey, 70 participants were randomly selected from the original population for a second survey and used a circadian rhythm assessment diary to record their meal times, bedtimes, and waking times for seven consecutive days. The mean ± SD of NFD at the first survey was 13.31 ± 1.00 h, and the mean NFD of the second survey was 13.59 ± 0.92 h, with no significant differences observed between the two surveys (*P* > 0.05). The test-retest correlation of NFD in the first and the second surveys was 0.74 (*P* < 0.01). The mean ± SD of NFD in the circadian rhythm assessment diary was 13.62 ± 1.03 h, and the correlation coefficient (*r*) between diary-reported and questionnaire-reported NFD was 0.71 (*P* < 0.01). Furthermore, Bland-Altman plot was used to assess the consistency between these two methods (Fig S1). The above results indicate that the questionnaire-reported NFD had good reliability and validity.

### Evaluation of healthy aging

Based on the framework proposed by Rowe and Kahn [28] and similar previous studies [29–31], healthy aging was defined as being free of major chronic diseases, no physical function limitation, no cognitive impairment, and no depressive symptoms.

#### Major chronic diseases

Participants were asked, “Have you ever been diagnosed with the following diseases by a hospital at or above the district level?” Major chronic diseases included diabetes, cardiovascular diseases (coronary heart disease), stroke, chronic lung diseases (bronchitis, emphysema, asthma) and cancer [29]. Participants reporting none of these conditions were classified as having no major chronic diseases.

#### Physical function

Physical function was assessed using the Physical Functioning (PF) subscale of the 36-Item Short Form Health Survey (SF-36), which has been validated in older adults in China (Cronbach’s alpha = 0.92, item discriminant validity = 0.49–0.81) [32]. The scale includes 10 items about physical activity limitations, with options divided into not limited, some limited, and very limited. Physical function limitation was defined as any of the following: at least “some limited” in moderate activities (moderate physical activities such as moving a table, sweeping the floor or doing exercises, climbing one floor, walking 100 m, walking 1000 m or walking more than 1500 m, bathing or dressing oneself), or “very limited” in more difficult activities (heavy physical activities such as running, lifting weights, strenuous exercise, carrying daily necessities such as buying groceries, shopping, climbing several floors, bending over, bending knees, squatting); otherwise, it was classified as no physical function limitation [33].

#### Cognitive function

Cognitive function was assessed using the Chinese version of the Mini-Mental State Examination (MMSE), which has been demonstrated to be reliable and valid among older adults in China (sensitivity = 83.87%, specificity = 84.48%, test-retest reliability = 0.75) [34]. The MMSE consists of 30 items that evaluate the following dimensions: orientation to time and place, attention and calculation, memory, recall, language ability, and executive function. Each correct response is scored as 1 point, while incorrect responses are scored as 0 points. The total score ranges from 0 to 30, with higher scores indicating better cognitive function. The thresholds for defining cognitive impairment were as follows: illiterate participants (no formal education) ≤ 17 points, primary education (≤ 6 years of schooling) ≤ 20 points, and secondary or higher education (> 6 years of schooling) ≤ 24 points [35].

#### Depressive symptoms

Depressive symptoms were assessed using the Chinese version of the 15-item Geriatric Depression Scale (GDS-15), which has demonstrated high reliability and validity in Asian older populations (sensitivity = 97%, specificity = 95%, Cronbach’s alpha = 0.80) [36]. The questionnaire consists of 15 items, 10 of which are positively scored (“yes” indicates the presence of depressive symptoms and is scored as 1 point), and 5 of which are negatively scored (“no” indicates the presence of depressive symptoms and is scored as 1 point). The higher the score, the more severe the depressive symptoms. Participants scoring < 6 points were classified as having no depressive symptoms [37].

### Assessment of covariates

Covariates were selected a priori based on previous literature and a conceptual framework and were grouped into four domains: demographic and socioeconomic characteristics, lifestyle and health status information, dietary behaviors, and circadian-related factors.

Demographic socioeconomic characteristics included age (continuous), sex (male, female), marital status (married, divorced/widowed/never married), community type (urban, rural), occupation (farmers, non-farmers), annual family income (< 20,000RMB, 20,000RMB-49,999RMB, ≥ 50,000RMB), education level (illiterate, primary and below, junior high and above).

Lifestyle and health status information included smoking status (never, former, current), drinking status (never, former, current), sedentary time (< 4 h, ≥ 4 and < 8 h, ≥8 h), body mass index (BMI) (< 24.00 kg/m^2^, ≥ 24.00 kg/m^2^), physical activity (low, middle, high). BMI was calculated by dividing weight (kg) by height squared (m²) and categorized into non-overweight (< 24.00 kg/m^2^) and overweight or above (≥ 24.00 kg/m^2^). Physical activity was measured using the Chinese version of the International Physical Activity Questionnaire-Short Form (IPAQ-SF), and then weekly metabolic equivalent of tasks (METs) minutes were calculated by multiplying the MET values of walking (3.3), moderate-intensity activities (4.0), and vigorous-intensity activities (8.0) by the respective durations (minutes) and frequencies (days per week). Participants were categorized into three physical activity levels: high, middle, and low [38].

Dietary behaviors included self-assessed satiety (1–10 points), number of eating occasions (continuous), and diet quality (low, high). Diet quality was evaluated using the validated quantitative food frequency questionnaire (FFQ). Each item on the questionnaire was scored on a five-point scale (daily consumption, 4–6 times per week, 1–3 times per week, several times per month, or rarely/never). Participants were awarded one point for each of the following criteria: daily consumption of fresh vegetables and fresh fruits, 1–6 times of red meat per week, ≥ 4 times of legumes per week, and ≥ 1 time of fish per week. The total score ranged from 0 to 5, with a score of 4 or higher indicating high quality diet and 3 or less indicating low diet quality [39].

Circadian-related factors included shift work experience (no, yes), nap rhythm (never, non-daily, daily), sleep duration (< 6 h, 6–8 h, >8 h), sleep quality (good, poor), and chronotype (intermediate, moderate morning, definite morning). Sleep quality was assessed by the Chinese version of the Pittsburgh Sleep Quality Index (PSQI). A PSQI score > 7 was classified as indicating poor sleep quality, while a score of 7 or less was classified as indicating good sleep quality [40]. We assessed chronotype by the reduced Morningness-Eveningness Questionnaire-5 (rMEQ-5). Chronotypes were categorized into five groups: definite evening type (4–7 points), moderate evening type (8–11 points), intermediate type (12–17 points), moderate morning type (18–21 points), and definite morning type (22–25 points) [41].

### Statistical analysis

Continuous variables were expressed as means ± standard deviations (SD) or medians (*P*_25_, *P*_75_), while categorical variables were presented as counts and percentages. Group comparisons were performed using one-way analysis of variance (ANOVA) or Kruskal-Wallis tests for continuous variables and Chi-squared tests for categorical variables. RCS was used to explore the potential nonlinear associations between nighttime fasting duration, breakfast time, dinner time, and healthy aging, as well as its four dimensions. Logistic regression models were performed to estimate odds ratios (OR) and 95% confidence intervals (CI) for the associations between NFD, breakfast time, dinner time, and healthy aging. We constructed a series of multivariable models with sequential adjustment. Model 1 adjusted for demographic and socioeconomic characteristics, lifestyle and health status information, which were considered core confounders. Model 2 additionally adjusted for dietary behaviors based on Model 1. Model 3 additionally adjusted for circadian-related factors based on Model 1. Model 4 included all of the above covariates. To examine the consistency of associations between NFD, breakfast time, dinner time, and healthy aging across key participant subgroups, stratified analyses were conducted by age, sex, education level, community type, smoking status, drinking status, BMI, physical activity, nap rhythm, sleep quality, and sleep duration, which are potential important confounders and commonly used stratification variables in epidemiological studies [30, 42, 43]. As these analyses were exploratory, no multiple testing corrections were applied. To verify the robustness of the results, we performed sensitivity analyses: first, restricted the sample to participants with specific characteristics, such as moderate morning or definite morning chronotypes, no shift work experience, and number of eating occasions was three and repeated the above analyses. Second, to strengthen the reliability of our results, E-values were calculated to estimate how strong an unmeasured confounder would be needed to eliminate the association found [44]. For some missing variables, we performed multiple imputation (R package, mice, 5) [45]. R 4.3.2 was used for all analyses and visualizations. Statistical significance was set at *P* < 0.05.

## Results

Among the 901 older adults included in the study, the median age was 69.00 years (IQR: 64.50, 74.00), and 56.60% were female. The average NFD was 13.30 ± 1.10 h, with 36.51% of participants (*n* = 329) having an NFD of 13 h to < 14 h. The breakfast time ranged from 4:00 to 9:30, with the majority (*n* = 520, 57.71%) having breakfast between 6:00 and 7:00. Dinner time ranged from 16:00 to 20:30, with most participants (*n* = 696, 77.25%) having dinner between 17:00 and 18:00.

In this study, 29.86% of participants (*n* = 269) achieved healthy aging. As for the four dimensions of healthy aging, 67.26% (*n* = 606) had no major chronic diseases, 43.06% (*n* = 388) had no physical limitations, 90.68% (*n* = 817) had no cognitive impairment, and 84.02% (*n* = 757) had no depressive symptoms.

### Basic characteristics of participants

Table 1 demonstrates the basic characteristics of participants across categories of NFD. Overall, significant differences were observed across NFD groups. Participants with shorter NFD (< 12 h) tended to be younger, have less sedentary time, higher levels of physical activity, higher reported satiety, a lower prevalence of a definite morning chronotype, earlier breakfast times, and later dinner times. Participants with longer NFD (≥ 15 h) tended to be older, have lower annual household incomes, higher levels of physical activity, a lower prevalence of a definite morning chronotype, longer sleep duration, later breakfast times, and earlier dinner times (all *P* < 0.05 from global tests across NFD groups).

**Table 1:** Basic characteristics of participants by nighttime fasting duration

Characteristics	Nighttime fasting duration, h					
	< 12	12 to < 13	13 to < 14	14 to < 15	≥ 15	*P-value*
	(*n* = 63)	(*n* = 189)	(*n* = 329)	(*n* = 244)	(*n* = 76)	
Age, *years*	67.00(62.00, 72.00)	68.00(64.00, 73.00)	69.00(65.00, 75.00)	70.00(65.00, 74.00)	71.00(66.00, 76.00)	0.002
Female, *n* (%)	37 (58.73)	105 (55.56)	181 (55.02)	144 (59.02)	43 (56.58)	0.892
Occupation, *n* (%)						0.314
Farmers	47 (74.60)	123 (65.08)	202 (61.40)	156 (63.93)	52 (68.42)	
Non-farmers	16 (25.40)	66 (34.92)	127 (38.60)	88 (36.07)	24 (31.58)	
Education level, *n* (%)						0.126
Illiterate	16 (25.40)	41 (21.69)	95 (28.88)	71 (29.10)	27 (35.53)	
Primary and below	24 (38.10)	95 (50.26)	127 (38.60)	106 (43.44)	32 (42.10)	
Junior high and above	23 (36.51)	53 (28.04)	107 (32.52)	67 (27.46)	17 (22.37)	
Community type, *n* (%)						0.373
Urban	7 (11.11)	34 (17.99)	63 (19.15)	38 (15.57)	17 (22.37)	
Rural	56 (88.89)	155 (82.01)	266 (80.85)	206 (84.43)	59 (77.63)	
Marital status, *n* (%)						0.076
Married	53 (84.13)	158 (83.60)	273 (82.98)	196 (80.33)	53 (69.74)	
Divorced/separated/widowed/never married	10 (15.87)	31 (16.40)	56 (17.02)	48 (19.67)	23 (30.26)	
Annual family income^1^, *n* (%)						< 0.001
< 20,000RMB	16 (25.40)	44 (23.28)	69 (20.97)	63 (25.82)	16 (21.05)	
20,000RMB-49,999RMB	32 (50.79)	77 (40.74)	151 (45.90)	135 (55.33)	55 (72.37)	
≥ 50,000RMB	15 (23.81)	66 (34.92)	109 (33.13)	46 (18.85)	5 (6.58)	
Sedentary time, *h*	4.00(2.00, 6.00)	4.50(2.50, 7.00)	5.00(3.50, 8.00)	5.00(3.00, 6.00)	5.00(3.00, 6.25)	0.003
Smoking status, *n* (%)						0.794
Never	41 (65.08)	135 (71.43)	249 (75.68)	178 (72.95)	57 (75.00)	
Former	10 (15.87)	25 (13.23)	36 (10.94)	33 (13.52)	7 (9.21)	
Current	12 (19.05)	29 (15.34)	44 (13.37)	33 (13.52)	12 (15.79)	
Drinking status ^1^, *n* (%)						0.093
Never	35 (55.56)	120 (63.49)	224 (68.09)	177 (72.54)	59 (77.63)	
Former	7 (11.11)	24 (12.70)	33 (10.03)	23 (9.43)	7 (9.21)	
Current	21 (33.33)	45 (23.81)	72 (21.88)	44 (18.03)	10 (13.16)	
Physical activity ^1^, *n* (%)						0.003
Low	7 (11.11)	34 (17.99)	70 (21.28)	28 (11.48)	14 (18.42)	
Middle	25 (39.68)	83 (43.92)	149 (45.29)	97 (39.75)	25 (32.89)	
High	31 (49.21)	72 (38.10)	110 (33.43)	119 (48.77)	37 (48.68)	
Body mass index ^1^, *kg/m*^*2*^	23.07(21.23, 25.73)	23.31(21.16, 25.35)	23.37(21.23, 25.16)	23.31(20.94, 25.48)	23.69(21.25, 25.28)	0.995
Sleep quality ^1^, *point*	5.00(3.00, 7.50)	4.00(3.00, 8.00)	5.00(3.00, 8.00)	5.00(3.00, 8.00)	5.00(3.00, 10.00)	0.502
Nap rhythm, *n* (%)						0.319
Never	30 (47.62)	74 (39.15)	123 (37.39)	95 (38.93)	33 (43.42)	
Non-daily	11 (17.46)	29 (15.34)	63 (19.15)	58 (23.77)	16 (21.05)	
Daily	22 (34.92)	86 (45.50)	143 (43.47)	91 (37.30)	27 (35.53)	
Had shift work experience ^1^, *n* (%)	3 (4.76)	29 (15.34)	50 (15.20)	42 (17.21)	11 (14.47)	0.187
Chronotype ^1^, *n* (%)						
Intermediate	4 (6.35)	16 (8.47)	21 (6.38)	29 (11.89)	8 (10.53)	< 0.001
Moderate morning	37 (58.73)	83 (43.92)	123 (37.39)	89 (36.48)	43 (56.58)	
Definite morning	22 (34.92)	90 (47.62)	185 (56.23)	126 (51.64)	25 (32.89)	
Sleep duration, *n* (%)						< 0.001
< 6 h	16 (25.40)	41 (21.69)	60 (18.24)	50 (20.49)	17 (22.37)	
6–8 h	41 (65.08)	124 (65.61)	221 (67.17)	132 (54.10)	27 (35.53)	
> 8 h	6 (9.52)	24 (12.70)	48 (14.59)	62 (25.41)	32 (42.11)	
Satiety ^1^, *points*	8.00(8.00, 10.00)	8.00(7.00, 9.00)	8.00(7.00, 8.00)	8.00(7.00, 8.00)	8.00(7.00, 8.00)	< 0.001
Diet quality^1^, *n* (%)						0.307
Low	55 (87.30)	164 (86.77)	274 (83.28)	195 (79.92)	61 (80.26)	
High	8 (12.70)	25 (13.23)	55 (16.72)	49 (20.08)	15 (19.74)	
Breakfast time^2^	5.54 ± 0.92	6.31 ± 0.63	6.76 ± 0.56	7.43 ± 0.51	8.14 ± 0.49	< 0.001
Dinner time^2^	18.61 ± 0.90	17.94 ± 0.64	17.50 ± 0.52	17.20 ± 0.47	16.94 ± 0.35	< 0.001
Number of eating occasions	3.10 ± 0.35	3.06 ± 0.28	3.09 ± 0.40	3.07 ± 0.34	3.01 ± 0.31	0.363

^1^ Missing data: Body Mass Index (*n* = 2,0.22%); shift work experience (*n* = 2, 0.22%); annual family income (*n* = 13, 1.44%); drinking status (*n* = 1, 0.11%); physical activity (*n* = 4, 0.44%); sleep quality (*n* = 8, 0.89%); chronotype (*n* = 9, 1.00%); satiety (*n* = 6, 0.67%); diet quality (*n* = 5, 0.5%)

^2^ Breakfast time and dinner time expressed as decimals, for example:5.50 means 5:30, 17.50 means 17:30

### Association between nighttime fasting duration and healthy aging

**Fig. 2:**
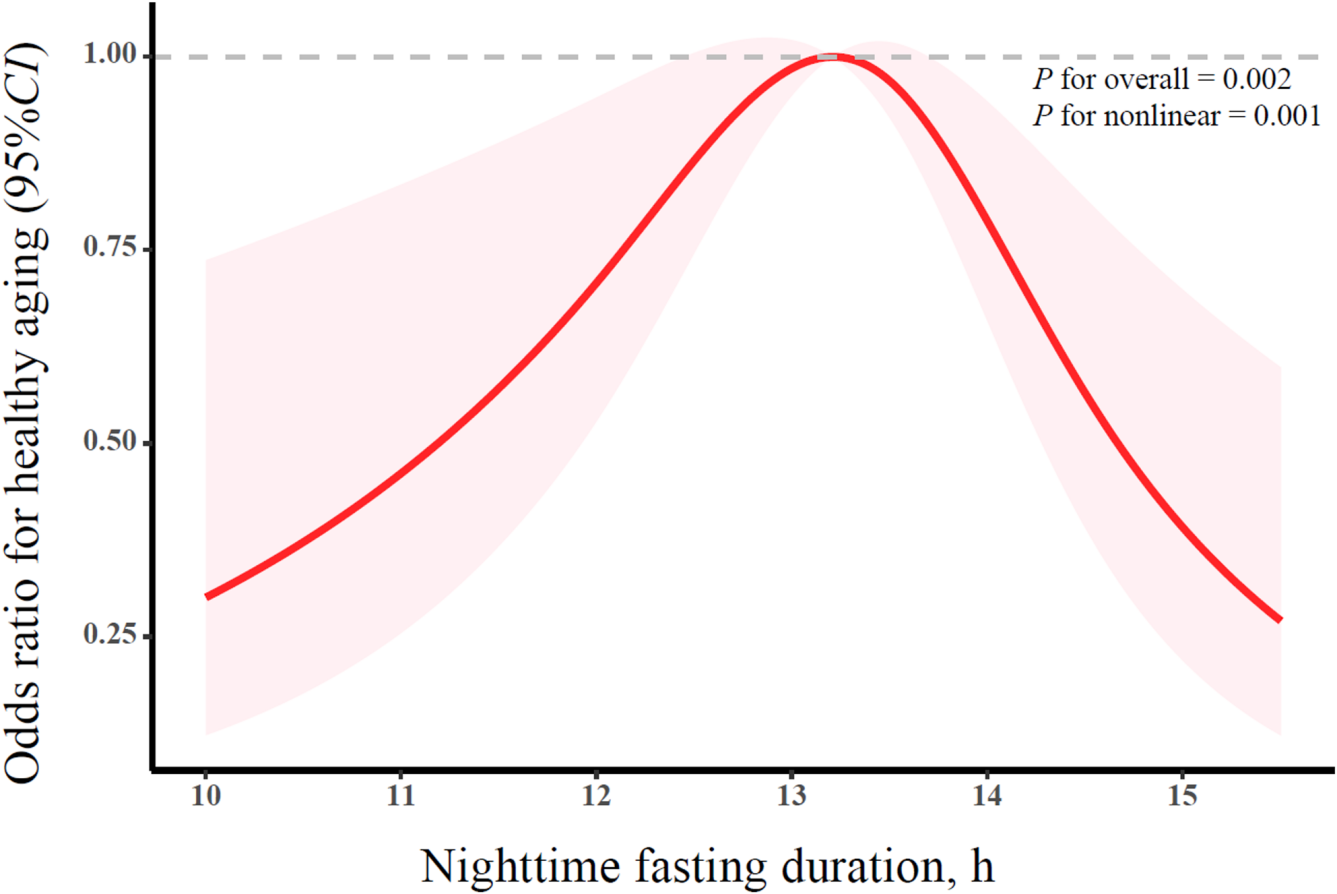
Nonlinear association between nighttime fasting duration and healthy aging among Chinese older adults (RCS analysis with covariates adjusted as in Model 4)

A preliminary exploration of the relationship between NFD and healthy aging using RCS revealed an inverted U-shaped association (*P*_overall_ = 0.002; *P*_nonlinear_ = 0.001) (Fig. 2). Table 2 shows the associations between NFD and healthy aging. After adjusting for basic demographic characteristics and lifestyle factors, compared to those with NFD of 13 h to < 14 h, participants with NFD of < 12 h, 14 h to < 15 h and ≥ 15 h were associated with lower odds of healthy aging (OR = 0.53, 95% CI: 0.28–1.02; OR = 0.64, 95% CI: 0.43–0.96; OR = 0.28, 95% CI: 0.12–0.62). Results remained substantially consistent after further adjustment for dietary behavior (Model 2) and circadian rhythm-related factors (Model 3). After adjustment for all covariates (Model 4), participants with NFD<12 h, 14 h to<15 h, and ≥ 15 h were significantly associated with lower odds of healthy aging (OR = 0.46, 95% CI: 0.23–0.93; OR = 0.62, 95% CI: 0.41–0.95; OR = 0.34, 95% CI: 0.15–0.77). The associations between NFD and the four dimensions of healthy aging are shown in Table S2.

**Table 2:** Relationship between NFD, breakfast time, dinner time and healthy aging

	*n* (%)		OR (95%CI)							
			Model 1		Model 2		Model 3			Model 4
**NFD**										
< 12 h	18(28.57)		0.53 (0.28–1.02)		**0.50 (0.25–0.97)**		**0.50 (0.25–0.98)**			**0.46 (0.23–0.93)**
12 h to < 13 h	68(35.98)		0.98 (0.65–1.48)		0.95 (0.63–1.44)		0.97 (0.64–1.49)			0.94 (0.61–1.44)
13 h to < 14 h	113(34.35)		1.00 (reference)		1.00 (reference)		1.00 (reference)			1.00 (reference)
14 h to < 15 h	62(25.41)		**0.64 (0.43–0.96)**		**0.63 (0.42–0.94)**		**0.63 (0.42–0.97)**			**0.62 (0.41–0.95)**
≥ 15 h	8(10.53)		**0.28 (0.12–0.62)**		**0.27 (0.12–0.61)**		**0.34 (0.15–0.78)**			**0.34 (0.15–0.77)**
**Breakfast time** ^**1**^										
< 6:00	23(29.11)		0.87 (0.50–1.52)		0.85 (0.48–1.48)		0.81 (0.45–1.43)			0.78 (0.44–1.40)
6:00–7:00	173(33.27)		1.00 (reference)		1.00 (reference)		1.00 (reference)			1.00 (reference)
> 7:00	73(24.17)		**0.62 (0.44–0.88)**		**0.62 (0.44–0.88)**		**0.67 (0.47–0.97)**			**0.68 (0.47–0.98)**
**Dinner time** ^**1**^										
< 17:00	10(11.90)		**0.40 (0.20–0.83)**		**0.40 (0.20–0.82)**		0.48 (0.23–1.01)			0.48 (0.23–1.00)
17:00–18:00	229(32.90)		1.00 (reference)		1.00 (reference)		1.00 (reference)			1.00 (reference)
> 18:00	30(24.79)		**0.49 (0.30–0.80)**		**0.48 (0.29–0.79)**		**0.50 (0.30–0.83)**			**0.49 (0.29–0.82)**

Bold means the results are statistically significant

^1^ The exact tertiles were < 6.50, 6.50–7.16, > 7.17 for breakfast time; <17.00, 17.00–17.99.00.99, > 17.99 for dinner time

Model 1: age (continuous); sex (male, female); occupation (farmer, no-farmer); community type (urban, rural); education level (illiterate, primary and below, junior high and above); annual family income (< 20,000RMB, 20,000RMB-49,999RMB, ≥ 50,000RMB); marital status (married, divorced/separated/widowed/never married); sedentary time (< 4 h, ≥ 4 and < 8 h, ≥8 h); smoking status (never, former, current); drinking status (never, former, current); physical activity (low, middle, high); BMI (< 24.00 kg/m^2^, ≥ 24.00 kg/m^2^)

Model 2: Model 1 + satiety (continuous); number of eating occasions (continuous); diet quality (low, high)

Model 3: Model 1 + shift work experience (no, yes); nap rhythm (never, non-daily, daily); sleep duration (< 6 h, 6–8 h, >8 h); sleep quality (good, poor); chronotype (intermediate, moderate morning, definite morning)

Model 4: all adjusted

The RCS showed different associations between NFD and the four dimensions of healthy aging: “no major chronic diseases” (*P*_overall_ = 0.175, *P*_nonlinear_ = 0.531); “no limitation of physical function” (*P*_overall_ = 0.028, *P*_nonlinear_ = 0.021); “no cognitive impairment” (*P*_overall_ = 0.001, *P*_nonlinear_ = 0.091); “no depressive symptoms” (*P*_overall_ = 0.273, *P*_nonlinear_ = 0.115) (Fig S2). Specifically, an inverted U-shaped association was observed between NFD and “no limitation of physical function”. Moreover, NFD was significantly associated with “no cognitive impairment”, with shorter NFD corresponding to lower odds of “no cognitive impairment”.

### Association between breakfast time, dinner time, and healthy aging

**Fig. 3:**
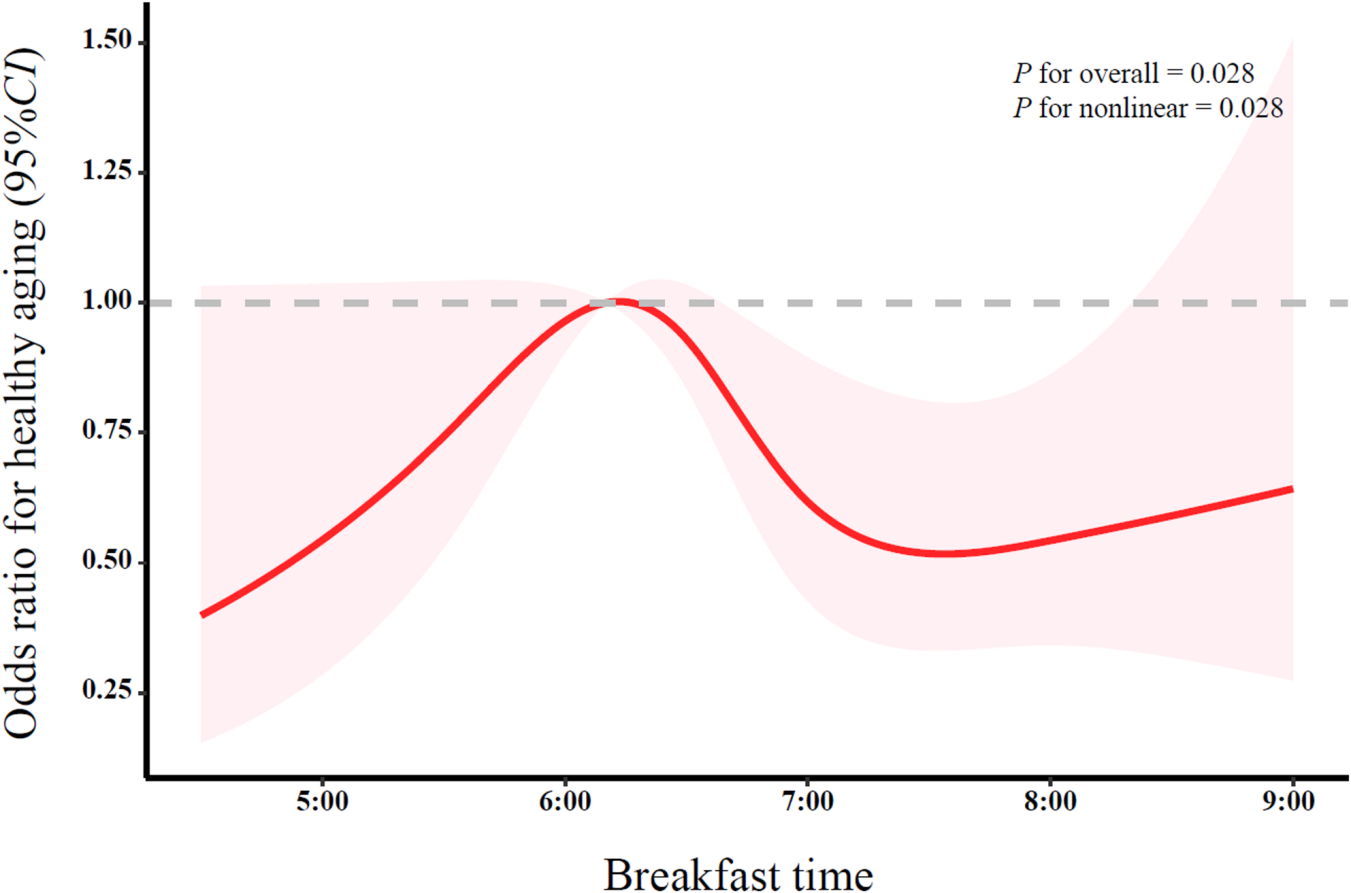
Nonlinear association between breakfast time and healthy aging among Chinese older adults (RCS analysis with covariates adjusted as in Model 4)

**Fig. 4:**
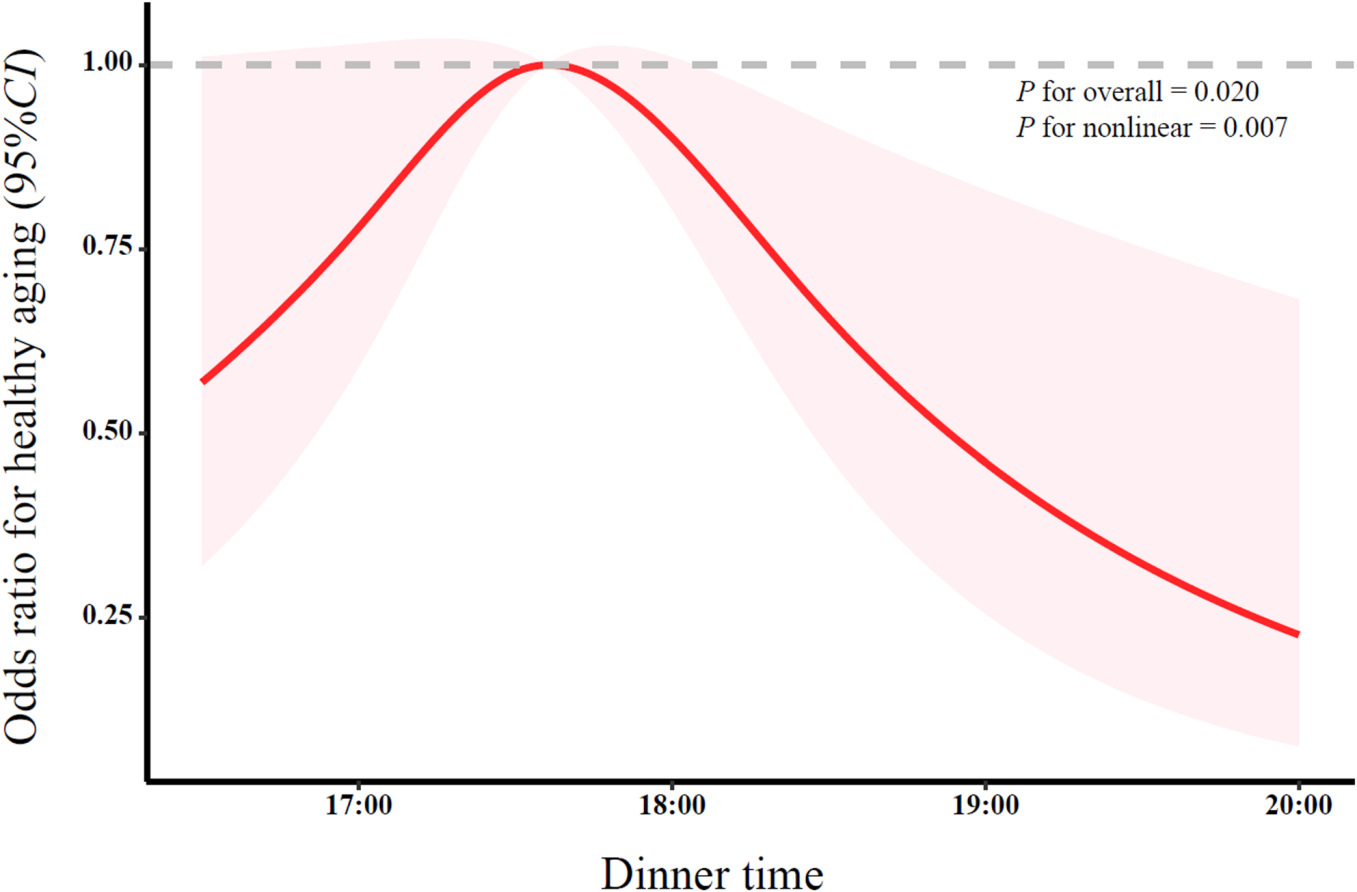
Nonlinear association between dinner time and healthy aging among Chinese older adults (RCS analysis with covariates adjusted as in Model 4)

Similarly, the RCS was used to explore the associations between breakfast time (*P*_overall_ = 0.028; *P*_nonlinear_ = 0.028), dinner time (*P*_overall_ = 0.020; *P*_nonlinear_ = 0.007) and healthy aging, indicating an inverted U-shaped association (Fig. 3 and Fig. 4). Table 2 presents the associations between breakfast time, dinner time and healthy aging. After adjusting for demographic socioeconomic characteristics, lifestyle and health status variables (Model 1), compared with participants who had breakfast at 6:00–7:00, those with early (< 6:00) and late (> 7:00) breakfast were associated with lower odds of healthy aging (OR = 0.87, 95% CI: 0.50–1.52; OR = 0.62, 95% CI: 0.44–0.88). Similarly, compared with those who had dinner at 17:00–18:00, participants with early (< 17:00) and late (> 18:00) dinner were associated with lower odds of healthy aging (OR = 0.40, 95% CI: 0.20–0.83; OR = 0.49, 95% CI: 0.30–0.80). Further adjustment for dietary behaviors (Model 2) and circadian-related factors (Model 3) yielded substantially consistent results. After full adjustment (Model 4), participants who had breakfast later (> 7:00) and dinner later (> 18:00) remained associated with lower odds of healthy aging (OR = 0.68, 95% CI: 0.47–0.98; OR = 0.49, 95% CI: 0.29–0.82). The associations between breakfast time, dinner time and the four dimensions of healthy aging are shown in Table S3.

The RCS showed different associations between breakfast time, dinner time, and the four dimensions of healthy aging. Later breakfast time was associated with lower odds of “no major chronic diseases” and higher odds of “no cognitive impairment”. However, breakfast time showed an inverted U-shaped association with “no limitation of physical function” (Fig S3). Dinner time also exhibited an inverted U-shaped association with “no limitation of physical function”; additionally, later dinner time was associated with lower odds of “no cognitive impairment” (Fig S4).

### Stratification and sensitivity analysis

First, stratified analyses by breakfast and dinner time showed that, overall, the association between NFD and healthy aging generally followed an inverted U-shaped relationship regardless of whether participants had normal or abnormal breakfast and dinner times, and no statistically significant interaction was observed (Fig. S5-S6).

Then we performed stratified analyses by age, sex, education level, community type, smoking status, drinking status, nap rhythm, BMI, physical activity levels, sleep quality, and sleep duration. The results indicated that the associations between NFD, breakfast time, dinner time, and healthy aging remained consistent across most subgroups. Interestingly, statistically significant interactions were observed between breakfast time and drinking status (*P*_interaction_ = 0.002), as well as BMI (*P*_interaction_ = 0.032). Specifically, among past and current drinkers, breakfast time < 6:00 or > 7:00 were associated with lower odds of achieving healthy aging (OR = 0.30, 95% CI: 0.09–0.97; OR = 0.27, 95% CI: 0.27–0.56). In addition, among participants with BMI < 24.0 kg/m², breakfast time > 7:00 was associated with lower odds of achieving healthy aging (OR = 0.47, 95% CI: 0.29–0.78) (Table S4-S6).

In addition, we performed some sensitivity analyses. Considering that the majority of older adults tend to have moderately or definitely morning chronotype, no shift work experience, follow a dietary pattern consisting of three main meals without snacks and maintain moderate to high levels of physical activity, we selected these populations for additional sensitivity analyses and found that the results were generally consistent with the primary results (Table S7-S9). Furthermore, E-value analyses suggested that relatively strong unmeasured confounding would be required to fully explain the observed associations: compared to 13 h ≤ NFD < 14 h, the E-values of the NFD < 12 h group, 14 h ≤ NFD < 15 h group, NFD ≥ 15 h group were 2.31 (1.23), 1.86 (1.19), and 2.82 (1.54) (Fig S7). These E-values indicate that an unmeasured confounder would need to be associated with both nighttime fasting duration and healthy aging by an association of at least this magnitude, comparable to or greater than those observed for the major measured confounders included in the fully adjusted model, to completely explain away the observed associations.

## Discussion

### Main findings

To our knowledge, this is the first study to investigate the association between NFD, the timing of breakfast and dinner, and healthy aging. We observed an inverted U-shaped relationship between NFD, meal timing, and healthy aging. Specifically, older adults with an NFD of 13 h to<14 h, breakfast between 6:00–7:00, and dinner between 17:00–18:00 had the highest odds of healthy aging. In contrast, shorter or longer nighttime fasting durations, as well as earlier or later meal timing, were associated with lower odds of healthy aging.

### Comparison with other studies

Most animal and human studies have found that extending NFD had positive health effects, particularly for metabolic health [46–49]. However, our study observed a nonlinear (inverted U-shaped) relationship: older adults with an NFD of < 12 h or > 14 h have lower odds of healthy aging. Our findings are consistent with several large population-based studies. For instance, a nationally representative prospective cohort study among 30,646 adults in the United States found that compared to those with an NFD of 10–11 h, individuals with an NFD ≤ 10 h or ≥ 14 h had higher risk of all-cause mortality, with hazard ratios of 1.23 (95% *CI*: 1.08–1.39) and 1.36 (95% *CI*: 1.19–1.54), respectively [15]. Another study among 10,561 older Americans reported a significant U-shaped relationship between NFD and both all-cause and cardiovascular disease (CVD) mortality, and found that an NFD of 11.49 h was associated with the lowest all-cause mortality, while an NFD of 7.35 h was linked to the lowest CVD mortality [50]. The latest research further found that shorter (< 10 h) or longer (> 14.1 h) NFD was associated with higher biological age markers [43]. In summary, these findings suggest that appropriate NFD is associated with lower mortality risk and more favorable bio-aging characteristics, and may be related to better overall health in older adults.

We further compared our findings for each dimension of healthy aging with prior studies. Regarding major chronic diseases, two analyses from the French NutriNet-Santé cohort reported that the NFD was not significantly associated with overall cardiovascular diseases and type 2 diabetes, while later timing of the first meal was associated with higher risks of both outcomes [16, 17]. Consistent with these findings, we also observed no clear association between NFD and the “no major chronic diseases” component, whereas later breakfast timing was associated with a lower likelihood of this component. For physical function, previous cross-sectional studies of older adults have shown that delayed first-meal times and prolonged nighttime fasting are associated with lower grip strength or lower limb function, and delayed last-meal times were associated with greater muscle mass [51, 52]. Almost in line with this evidence, our study found that later breakfast timing and earlier dinner timing were both associated with a lower likelihood of “no limitation of physical function”, whereas the association with longer NFD lost statistical significance after full adjustment, which may be attributable to differences in physical function measurement methods across studies. With respect to cognitive outcomes, an Italian study reported a lower likelihood of cognitive impairment among individuals with shorter eating windows [53]. Similarly, in our analysis, shorter nighttime fasting duration was associated with a lower likelihood of “no cognitive impairment”, consistent with the observed association between later dinner times and a lower likelihood of “no cognitive impairment”. For mental health, a large study based on the National Health and Nutrition Examination Survey reported a U-shaped association between NFD and depression, with both shorter and longer NFD associated with higher odds of depression [54]. In contrast, we did not observe a statistically significant nonlinear association between NFD and the “no depressive symptoms” component; however, the direction of the estimates was generally consistent with the main findings. This may partly reflect limited statistical power and differences in outcome definitions.

### Potential mechanism

NFD and meal timing may influence healthy aging through regulation of the circadian rhythm system. The circadian rhythm is driven by a cell-autonomous negative feedback loop, primarily orchestrated by the transcription activators circadian locomotor output cycles kaput (and its homolog neuronal PAS domain protein 2) and muscle ARNT-like protein 1 [55]. This system is present in virtually all human cells and regulates a wide range of physiological functions, including sleep, metabolism, and immunity. Meal timing, as a crucial external cue for circadian rhythms, effectively regulates the biological clock of peripheral tissues [9]. When meal time deviate from the optimal time window of the circadian rhythm, circadian disruption may occur, leading to an increased risk of metabolic, cardiovascular, neurological, and mood disorders [56], as well as impaired skeletal muscle health [57]. Furthermore, meal timing can influence the gut microbiota, which, via the gut-brain axis, impacts cognitive function and mood regulation [58].

At the same time, during fasting, glycogen stores are depleted, and fatty acid metabolism generates ketone bodies such as acetoacetate (AcAc) and beta-hydroxybutyrate (BHB). These ketone bodies can upregulate brain-derived neurotrophic factor (BDNF), a critical factor in maintaining neuronal health and cognitive function [59]. Fasting also reduces the levels of pro-inflammatory cytokines (e.g., IL-6 and TNF-α), modulates oxidative stress responses, and decreases chronic inflammation [49]. However, in older adults—who are at increased risk of inadequate energy and nutrient intake—excessively prolonged fasting or poorly timed meals may compromise nutritional adequacy. Appropriately timed eating patterns may therefore help balance circadian alignment with sufficient nutrient intake, supporting metabolic regulation, muscle maintenance, and cognitive health, and ultimately contributing to healthy aging.

### Strengths and limitations

The strength of this study is that it explores the association between NFD, meal timing (breakfast and dinner), and healthy aging specifically in a Chinese elderly population. Additionally, this study considered the potential confounding effects of circadian rhythm factors, such as chronotype, sleep quality, and napping patterns, on the relationship between NFD, meal timing, and healthy aging. This consideration enhances the reliability of our findings to a certain extent. However, this study also has some limitations. First, as a cross-sectional study, it does not allow for causal inferences. Longitudinal studies are necessary in the future to further validate our conclusions. However, the inclusion of participants from regions with varying economic conditions across China enhances the representativeness of our study population to a certain degree. Second, NFD and meal timing were based on self-reported data, which may have introduced information bias. Recall bias may lead to misclassification, likely in a non-differential manner (i.e., unrelated to healthy aging status). Such non-differential misclassification of exposure tends to bias effect estimates toward the null, potentially attenuating the observed odds ratios and making our reported associations conservative underestimates of the true effects. More importantly, if social desirability bias exists (e.g., healthier individuals more “ideally” report their habits), it could influence both the magnitude and shape of the observed U-shaped relationship. This underscores that the identified “optimal” windows (NFD of 13 h to <14 h, etc.) should be interpreted as preliminary associations. While we attempted to mitigate these issues by using trained interviewers and standardized protocols, residual bias cannot be ruled out. Future studies employing objective measures of eating timing (e.g., timestamps from wearable devices) are needed to confirm these associations. However, the validation results of the dietary behavior questionnaire showed that self-reported NFD had good reliability and validity. Third, although we adjusted for a wide range of confounding factors, the influence of residual confounding cannot be completely ruled out. To address this, we calculated E-values to evaluate the potential impact of unmeasured confounders, which further reinforced the robustness of our findings. It should also be noted that some covariates, particularly dietary behaviors and circadian-related factors, are closely related to eating time behaviors and may partially lie on the causal pathway under certain assumptions. Therefore, estimates from the fully adjusted models should be interpreted as associations conditional on these factors rather than total effects. Fourth, our study did not dynamically monitor or investigate changes in eating/fasting habits over time, limiting our ability to assess their dynamic relationship with health outcomes. Lastly, as our participants were drawn exclusively from a Chinese elderly population, the findings may not be generalizable to other countries or ethnic groups.

## Conclusion

In this sample of Chinese older adults, eating time behaviors, including nighttime fasting duration, breakfast time, and dinner time, exhibited nonlinear associations with healthy aging. Optimal circadian-related eating patterns, specifically a nighttime fasting duration of 13h to < 14 h, breakfast between 6:00 and 7:00, and dinner between 17:00 and 18:00, were associated with the highest odds of healthy aging, and these associations were generally consistent across different subgroups. These findings provide new insights into the potential role of chrononutrition in healthy aging among older adults. Further confirmation in prospective cohort studies is warranted.

## Supplementary Information

Supplementary Material 1

Supplementary Material 2. Table S1 Comparison of characteristics of excluded and included individuals. Table S2 Relationship between NFD and four dimensions of healthy aging. Table S3 Relationship between breakfast time, dinner time and four dimensions of healthy aging. Table S4 Stratified analysis for nighttime fasting duration and healthy aging. Table S5 Stratified analysis for breakfast time and healthy aging. Table S6 Stratified analysis for dinner time and healthy aging. Table S7 Odds ratio of healthy aging by nighttime fasting duration, sensitivity analysis. Table S8 Odds ratio of healthy aging by breakfast time, sensitivity analysis. Table S9 Odds ratio of healthy aging by dinner time, sensitivity analysis. Fig S1 Bland-Altman plot of diary-reported and questionnaire-reported NFD. Fig S2 RCS analyses of NFD in relation to the four dimensions of healthy aging (all the same covariates as in Model 4 were adjusted). Fig S3 RCS analyses of breakfast time in relation to the four dimensions of healthy aging (all the same covariates as in Model 4 were adjusted). Fig S4 RCS analyses of dinner time in relation to the four dimensions of healthy aging (all the same covariates as in Model 4 were adjusted). Fig S5 RCS analyses of NFD and healthy aging according to breakfast time: (A) normal breakfast time (6:00–7:00) and (B) abnormal breakfast time (<6:00 or >7:00), adjusted for the same covariates as in Model 4. Fig S6 RCS analyses of NFD and healthy aging according to dinner timing: (A) normal dinner time (6:00–7:00) and (B) abnormal dinner time (<6:00 or >7:00), adjusted for the same covariates as in Model 4. Fig S7 Curves of the sensitivity analysis for unobserved confounders with E-value highlighted

## Data Availability

The data presented in this study are available on request from the corresponding authors.

## Abbreviations

NFD: Nighttime fasting duration
PF: Physical Functioning
SF-36: 36-Item Short Form Health Survey
MMSE: Mini-Mental State Examination
GDS-15: 15-item Geriatric Depression Scale
BMI: Body mass index
FFQ: Food frequency questionnaire
IPAQ-SF: International Physical Activity Questionnaire-Short Form
METs: Metabolic equivalent of tasks
PSQI: Pittsburgh Sleep Quality Index
rMEQ-5: Reduced Morningness-Eveningness Questionnaire-5
SD: Standard deviations
OR: Odds ratios
CI: Confidence intervals
ANOVA: Analysis of variance
RCS: Restricted cubic splines
AcAc: Acetoacetate
BHB: Beta-hydroxybutyrate
BDNF: Brain-derived neurotrophic factor
