# Supplementary material for: Association of nighttime fasting duration, breakfast time and dinner time with healthy aging in Chinese older adults: a cross‑sectional study
Source: Nutr J. 2026 Mar 23;25:55. doi: 10.1186/s12937-026-01317-7 (PMC13130698; doi:10.1186/s12937-026-01317-7)
Supplement: Supplementary file 2 — Supplementary Material 2. Table S1 Comparison of characteristics of excluded and included individuals. Table S2 Relationship between NFD and four dimensions of healthy aging. Table S3 Relationship between breakfast time, dinner time and four dimensions of healthy aging. Table S4 Stratified analysis for nighttime fasting duration and healthy aging. Table S5 Stratified analysis for breakfast time and healthy aging. Table S6 Stratified analysis for dinner time and healthy aging. Table S7 Odds ratio of healthy aging by nighttime fasting duration, sensitivity analysis. Table S8 Odds ratio of healthy aging by breakfast time, sensitivity analysis. Table S9 Odds ratio of healthy aging by dinner time, sensitivity analysis. Fig S1 Bland-Altman plot of diary-reported and questionnaire-reported NFD. Fig S2 RCS analyses of NFD in relation to the four dimensions of healthy aging (all the same covariates as in Model 4 were adjusted). Fig S3 RCS analyses of breakfast time in relation to the four dimensions of healthy aging (all the same covariates as in Model 4 were adjusted). Fig S4 RCS analyses of dinner time in relation to the four dimensions of healthy aging (all the same covariates as in Model 4 were adjusted). Fig S5 RCS analyses of NFD and healthy aging according to breakfast time: (A) normal breakfast time (6:00–7:00) and (B) abnormal breakfast time (<6:00 or >7:00), adjusted for the same covariates as in Model 4. Fig S6 RCS analyses of NFD and healthy aging according to dinner timing: (A) normal dinner time (6:00–7:00) and (B) abnormal dinner time (<6:00 or >7:00), adjusted for the same covariates as in Model 4. Fig S7 Curves of the sensitivity analysis for unobserved confounders with E-value highlighted [file 12937_2026_1317_MOESM2_ESM.docx]

**Supplementary Material1**

**Association of nighttime fasting duration, breakfast time and dinner time with healthy aging in Chinese older adults: a cross‑sectional study**

**Table S1** Comparison of characteristics of excluded and included individuals

**Table S2** Relationship between NFD and four dimensions of healthy aging

**Table S3** Relationship between breakfast time, dinner time and four dimensions of healthy aging

**Table S4** Stratified analysis for nighttime fasting duration and healthy aging

.

**Table S5** Stratified analysis for breakfast time and healthy aging

**Table S6** Stratified analysis for dinner time and healthy aging

**Table S7** Odds ratio of healthy aging by nighttime fasting duration, sensitivity analysis

**Table S8** Odds ratio of healthy aging by breakfast time, sensitivity analysis

**Table S9** Odds ratio of healthy aging by dinner time, sensitivity analysis

**Fig S1** Bland-Altman plot of diary-reported and questionnaire-reported NFD

**Fig S2** RCS analyses of NFD in relation to the four dimensions of healthy aging (all the same covariates as in Model 4 were adjusted)

**Fig S3** RCS analyses of breakfast time in relation to the four dimensions of healthy aging (all the same covariates as in Model 4 were adjusted)

**Fig S4** RCS analyses of dinner time in relation to the four dimensions of healthy aging (all the same covariates as in Model 4 were adjusted)

**Fig S5** RCS analyses of NFD and healthy aging according to breakfast time: (A) normal breakfast time (6:00–7:00) and (B) abnormal breakfast time (<6:00 or >7:00), adjusted for the same covariates as in Model 4

**Fig S6** RCS analyses of NFD and healthy aging according to dinner timing: (A) normal dinner time (6:00–7:00) and (B) abnormal dinner time (<6:00 or >7:00), adjusted for the same covariates as in Model 4

**Fig S7** Curves of the sensitivity analysis for unobserved confounders with E-value highlighted

**Table S1** Comparison of characteristics of excluded and included subjects

|  | excluded | included | *P*-value |
| --- | --- | --- | --- |
|  | N=46 | N=901 |  |
| **Age, *years*** | 69.00 (67.00-74.00) | 69.00 (65.00-74.00) | 0.303 |
| **Female, *n* (%)** | 28 (60.87) | 510 (56.60) | 0.569 |
| **Occupation, *n* (%)** |  |  | **0.048** |
| Farmers | 23 (50.00) | 580 (64.37) |  |
| No-farmers | 23 (50.00) | 321 (35.63) |  |
| **Educational level,** ***n* (%)** |  |  | 0.138 |
| illiteracy | 19 (41.30) | 250 (27.75) |  |
| Primary school and below | 16 (34.78) | 384 (42.62) |  |
| Junior high school and above | 11 (23.91) | 267 (29.63) |  |
| **Community type, *n* (%)** |  |  | 0.236 |
| Urban | 5 (10.87) | 159 (17.65%) |  |
| Rural | 41 (89.13) | 742 (82.35%) |  |
| **Marital status, *n* (%)** |  |  | **0.048** |
| Married | 32 (69.57) | 733 (81.35) |  |
| Divorced/separated/widowed/ never married | 14 (30.43) | 168 (18.65) |  |
| **Annual family income, *n* (%)** |  |  | 0.095 |
| <20000RMB | 13 (38.24) | 204 (22.97) |  |
| 20000RMB-49999RMB | 12 (35.29) | 446 (50.23) |  |
| ≥50000RMB | 9 (26.47) | 238 (26.80) |  |
| **Sedentary time, *h*** | 5.00 (3.62-8.00) | 5.00 (3.00-7.00) | 0.430 |
| **Smoking status, *n* (%)** |  |  | 0.881 |
| Never | 30 (69.77) | 660 (73.25) |  |
| Former | 6 (13.95) | 111 (12.32) |  |
| Current | 7 (16.28) | 130 (14.43) |  |
| **Drinking status, *n* (%)** |  |  | 0.808 |
| Never | 28 (63.64) | 614 (68.22) |  |
| Former | 5 (11.36) | 94 (10.44) |  |
| Current | 11 (25.00) | 192 (21.33) |  |
| **Physical activity, *n* (%)** |  |  | 0.144 |
| Low | 12 (26.09) | 153 (17.06) |  |
| Middle | 21 (45.65) | 378 (42.14) |  |
| High | 13 (28.26) | 366 (40.80) |  |
| **Body Mass Index, *kg/m^2^*** | 22.63 (20.31-24.20) | 23.37 (21.23-25.39) | 0.183 |
| **Sleep quality, *n* (%)** |  |  | 0.078 |
| Good | 27 (58.70) | 633 (70.88) |  |
| Poor | 19 (41.30) | 260 (29.12) |  |
| **Sleep duration, h** |  |  | 0.237 |
| <6h | 13 (28.26) | 184 (20.42) |  |
| 6h-8h | 28 (60.87) | 545 (60.49) |  |
| >8h | 5 (10.87) | 172 (19.09) |  |
| **Nap rhythm, *n* (%)** |  |  | **0.015** |
| Never | 27 (58.70) | 355 (39.40) |  |
| Non-daily | 3 (6.52) | 177 (19.64) |  |
| Daily | 16 (34.78) | 369 (40.95) |  |
| **Shift work experience, *n* (%)** |  |  | 0.440 |
| No | 41 (89.13) | 764 (84.98) |  |
| Yes | 5 (10.87) | 135 (15.02) |  |
| **Chronotype, *n* (%)** |  |  | **0.033** |
| Intermediate | 9 (19.57) | 78 (8.74) |  |
| Moderate morning | 14 (30.43) | 370 (41.48) |  |
| Definite morning | 23 (50.00) | 444 (49.78) |  |

**Table S2** Relationship between NFD and four dimensions of healthy aging

|  |  | | |  | | ***OR* (*95%CI*)** | |  | |  |
| --- | --- | --- | --- | --- | --- | --- | --- | --- | --- | --- |
| Outcomes | NFD<12h | | | 12h≤NFD<13h | | 13h≤NFD<14h | | 14h≤NFD<15h | | NFD≥15h |
| **No major chronic diseases** | |  | | |  | | | |  | |
| *n* (%) | 47(74.60) | | | 136 (71.96) | | 222(67.48) | | 158 (64.75) | | 4343 (56.58) |
| Model 1 | 1.34 (0.70, 2.57) | | | 1.20 (0.79, 1.80) | | 1.00(reference) | | 0.87 (0.60, 1.26) | | 0.63 (0.39, 1.09) |
| Model 2 | 1.33 (0.69, 2.57) | | | 1.17 (0.78, 1.77) | | 1.00(reference) | | 0.88 (0.60, 1.27) | | 0.63 (0.37, 1.09) |
| Model 3 | 1.27 (0.65, 2.49) | | | 1.18 (0.78, 1.80) | | 1.00(reference) | | 0.88 (0.60, 1.28) | | 0.67 (0.38, 1.17) |
| Model 4 | 1.28 (0.65, 2.52) | | | 1.16 (0.74, 1.77) | | 1.00(reference) | | 0.89 (0.61, 1.30) | | 0.67 (0.38, 1.19) |
| **No limitation of physical function** | | |  | | | |  | | | |
| *n* (%) | 32(50.79) | | | 96(50.79) | | 147(44.68) | | 93(38.11) | | 20(26.32) |
| Model 1 | 0.94 (0.52, 1.71) | | | 1.15 (0.78, 1.72) | | 1.00(reference) | | 0.71 (0.49, 1.04) | | **0.52 (0.28, 0.95)** |
| Model 2 | 0.87 (0.47, 1.59) | | | 1.13 (0.76, 1.69) | | 1.00(reference) | | 0.70 (0.48, 1.02) | | **0.53 (0.29, 0.97)** |
| Model 3 | 0.94 (0.51, 1.73) | | | 1.15 (0.77, 1.72) | | 1.00(reference) | | 0.74 (0.50, 1.09) | | 0.60 (0.32, 1.13) |
| Model 4 | 0.86 (0.46, 1.59) | | | 1.12 (0.74, 1.68) | | 1.00(reference) | | 0.72 (0.49, 1.06) | | 0.61 (0.32, 1.14) |
| **No cognitive impairment** |  | | |  | |  | |  | |  |
| *n* (%) | 48(76.19) | | | 173(91.53) | | 300(91.19) | | 230(94.26) | | 66(86.84) |
| Model 1 | **0.18 (0.08, 0.41)** | | | 0.80 (0.39, 1.63) | | 1.00(reference) | | 1.66 (0.82, 3.38) | | 0.87 (0.37, 2.04) |
| Model 2 | **0.17 (0.07, 0.40)** | | | 0.80 (0.39, 1.64) | | 1.00(reference) | | 1.56 (0.76, 3.19) | | 0.80 (0.34, 1.90) |
| Model 3 | **0.19 (0.08, 0.46)** | | | 0.86 (0.41, 1.80) | | 1.00(reference) | | 2.05 (0.97, 4.30) | | 1.32 (0.53, 3.25) |
| Model 4 | **0.19 (0.08, 0.45)** | | | 0.88 (0.42, 1.85) | | 1.00(reference) | | 1.91 (0.90, 4.03) | | 1.26 (0.51, 3.14) |
| **No depressive symptoms** | |  | | |  | | | |  | |
| *n* (%) | 50(79.37) | | | 157(83.07) | | 287(87.23) | | 205(84.02) | | 5858 (76.32) |
| Model 1 | 0.47 (0.22, 1.00) | | | 0.65 (0.38, 1.12) | | 1.00(reference) | | 0.79 (0.48, 1.30) | | 0.52 (0.27, 1.02) |
| Model 2 | 0.47 (0.21, 1.01) | | | 0.67 (0.39, 1.15) | | 1.00(reference) | | 0.79 (0.48, 1.31) | | 0.54 (0.27, 1.05) |
| Model 3 | 0.46 (0.21, 1.04) | | | 0.66 (0.37, 1.16) | | 1.00(reference) | | 0.87 (0.51, 1.48) | | 0.70 (0.34, 1.42) |
| Model 4 | 0.45 (0.20, 1.03) | | | 0.66 (0.37, 1.17) | | 1.00(reference) | | 0.86 (0.50, 1.47) | | 0.70 (0.34, 1.44) |

Bold means the results are statistically significant.

Model 1: age (continuous); sex (male, female); occupation (farmer, no-farmer); community type (urban, rural); education level (illiterate, primary and below, junior high and above); annual family income (<20,000RMB, 20,000RMB-49,999RMB, ≥50,000RMB); marital status (married, divorced/separated/widowed/never married); sedentary time (<4h, ≥4 and <8 h, ≥8h); smoking status (never, former, current); drinking status (never, former, current); physical activity (low, middle, high); BMI (<24.00 kg/m^2^, ≥24.00 kg/m^2^).

Model 2: model 1 + satiety (continuous); number of eating occasions (continuous); diet quality (low, high).

Model 3: model 1 + shift work experience (no, yes); nap rhythm (never, non-daily, daily); sleep duration (<6h, 6h-8h, >8h); sleep quality (good, poor); chronotype (intermediate, moderate morning, definite morning).

Model 4: all adjusted.

**Table S3** Relationship between breakfast time, dinner time and four dimensions of healthy aging

|  |  | **Breakfast time^1^** |  |  |  | **Dinner time^1^** |  |
| --- | --- | --- | --- | --- | --- | --- | --- |
| Outcomes | < 6:00 | 6:00-7:00 | > 7:00 |  | <17:00 | 17:00-18:00 | > 18:00 |
| **No major chronic diseases** | |  |  |  |  |  |  |
| *n* (%) | 59(74.68) | 362(69.62) | 185(61.26) |  | 53(63.10) | 476 (68.39) | 77(63.64) |
| Model 1 | 1.27 (0.72, 2.23) | 1.00 (reference) | **0.68 (0.49, 0.93)** |  | 0.92 (0.56, 1.53) | 1.00 (reference) | 0.73 (0.47, 1.13) |
| Model 2 | 1.26 (0.71, 2.21) | 1.00 (reference) | **0.68 (0.50, 0.93)** |  | 0.91 (0.55, 1.52) | 1.00 (reference) | 0.72 (0.46, 1.11) |
| Model 3 | 1.18 (0.66, 2.10) | 1.00 (reference) | **0.70 (0.51, 0.98)** |  | 1.02 (0.61, 1.70) | 1.00 (reference) | 0.77 (0.49, 1.20) |
| Model 4 | 1.17 (0.66, 2.09) | 1.00 (reference) | **0.71 (0.51, 0.99)** |  | 1.01 (0.60, 1.69) | 1.00 (reference) | 0.75 (0.48, 1.18) |
| **No limitation of physical function** | | |  |  |  |  |  |
| *n* (%) | 33(41.77) | 264(47.31) | 109(36.09) |  | 14(16.67) | 322(46.26) | 52(42.98) |
| Model 1 | 0.85 (0.51, 1.43) | 1.00 (reference) | **0.57 (0.42, 0.79)** |  | **0.33 (0.17, 0.62)** | 1.00 (reference) | 0.67 (0.43, 1.04) |
| Model 2 | 0.82 (0.49, 1.39) | 1.00 (reference) | **0.58 (0.42, 0.80)** |  | **0.33 (0.17, 0.61)** | 1.00 (reference) | 0.65 (0.42, 1.02) |
| Model 3 | 0.81 (0.48, 1.38) | 1.00 (reference) | **0.62 (0.45, 0.87)** |  | **0.35 (0.18, 0.67)** | 1.00 (reference) | 0.67 (0.42, 1.05) |
| Model 4 | 0.78 (0.45, 1.33) | 1.00 (reference) | **0.63 (0.45, 0.88)** |  | **0.35 (0.18, 0.66)** | 1.00 (reference) | 0.65 (0.41, 1.03) |
| **No cognitive impairment** | |  |  |  |  |  |  |
| *n* (%) | 69(87.34) | 471(90.58) | 277(91.72) |  | 76(90.48) | 641(92.10) | 100(82.64) |
| Model 1 | 0.66 (0.29, 1.47) | 1.00 (reference) | 1.38 (0.80, 2.38) |  | 1.16 (0.49, 2.73) | 1.00 (reference) | **0.39 (0.20, 0.73)** |
| Model 2 | 0.65 (0.29, 1.46) | 1.00 (reference) | 1.31 (0.76, 2.28) |  | 1.20 (0.51, 2.85) | 1.00 (reference) | **0.39 (0.20, 0.74)** |
| Model 3 | 0.63 (0.27, 1.46) | 1.00 (reference) | **1.80 (1.00, 3.23)** |  | 1.68 (0.68, 4.13) | 1.00 (reference) | **0.39 (0.20, 0.77)** |
| Model 4 | 0.63 (0.27, 1.46) | 1.00 (reference) | 1.76 (0.98, 3.17) |  | 1.75 (0.70, 4.33) | 1.00 (reference) | **0.40 (0.20, 0.80)** |
| **No depressive symptoms** | |  |  |  |  |  |  |
| *n* (%) | 64(81.01) | 452(86.92) | 241(79.80) |  | 64(76.19) | 599 (86.06) | 94(77.69) |
| Model 1 | 0.67 (0.35, 1.30) | 1.00 (reference) | **0.61 (0.41, 0.91)** |  | 0.57 (0.31, 1.02) | 1.00 (reference) | 0.61 (0.36, 1.04) |
| Model 2 | 0.68 (0.35, 1.31) | 1.00 (reference) | **0.61 (0.41, 0.92)** |  | 0.57 (0.32, 1.04) | 1.00 (reference) | 0.62 (0.36, 1.06) |
| Model 3 | 0.65 (0.32, 1.30) | 1.00 (reference) | 0.78 (0.50, 1.20) |  | 0.69 (0.37, 1.29) | 1.00 (reference) | 0.70 (0.38, 1.32) |
| Model 4 | 0.65 (0.32, 1.30) | 1.00 (reference) | 0.77 (0.50, 1.20) |  | 0.70 (0.39, 1.25) | 1.00 (reference) | 0.71 (0.40, 1.28) |

Bold means the results are statistically significant.

^1^ The exact tertiles were <6.50, 6.50-7.16, >7.17 for breakfast time; <17.00, 17.00-17.99, >17.99 for dinner time.

Model 1: age (continuous); sex (male, female); occupation (farmer, no-farmer); community type (urban, rural); education level (illiterate, primary and below, junior high and above); annual family income (<20,000RMB, 20,000RMB-49,999RMB, ≥50,000RMB); marital status (married, divorced/separated/widowed/never married); sedentary time (<4h, ≥4 and <8 h, ≥8h); smoking status (never, former, current); drinking status (never, former, current); physical activity (low, middle, high); BMI (<24.00 kg/m^2^, ≥24.00 kg/m^2^).

Model 2: model 1 + satiety (continuous); number of eating occasions (continuous); diet quality (low, high).

Model 3: model 1 + shift work experience (no, yes); nap rhythm (never, non-daily, daily); sleep duration (<6h, 6h-8h, >8h); sleep quality (good, poor); chronotype (intermediate, moderate morning, definite morning).

Model 4: all adjusted.

**Table S4** Stratified analysis for nighttime fasting duration and healthy aging

|  |  |  |  | *OR* (95%*CI*) * |  |  |  |
| --- | --- | --- | --- | --- | --- | --- | --- |
| Subgroup | **N** | ＜12h | 12h to＜13h | 13h to＜14h | 14h to＜15h | ≥15h | ***P* _interaction_** |
| **Age** |  |  |  |  |  |  | 0.601 |
| ≤70 years | 507 | 0.52 (0.23, 1.18) | 1.12 (0.66, 1.92) | 1.00 (reference) | 0.77 (0.45, 1.31) | **0.26 (0.08, 0.82)** |  |
| >70 years | 394 | 0.25 (0.05, 1.13) | 0.66 (0.29, 1.51) | 1.00 (reference) | **0.39 (0.18, 0.84)** | 0.32 (0.08, 1.23) |  |
| **Sex** |  |  |  |  |  |  | 0.248 |
| Male | 391 | 0.91 (0.33, 2.53) | 0.90 (0.47, 1.72) | 1.00 (reference) | 0.88 (0.47, 1.65) | **0.27 (0.08, 0.92)** |  |
| Female | 510 | **0.19 (0.06, 0.61)** | 0.95 (0.51, 1.76) | 1.00 (reference) | **0.45 (0.24, 0.83)** | 0.32 (0.10, 1.05) |  |
| **Education level** |  |  |  |  |  |  | 0.925 |
| Illiteracy | 250 | 0.22 (0.03, 1.43) | 1.00 (0.33, 3.04) | 1.00 (reference) | 0.71 (0.27, 1.86) | 0.14 (0.01, 1.33) |  |
| Primary and below | 384 | 0.45 (0.14, 1.51) | 0.69 (0.34, 1.41) | 1.00 (reference) | 0.48 (0.23, 1.01) | 0.37 (0.10, 1.39) |  |
| Junior high and above | 267 | 0.58 (0.18, 1.86) | 0.94 (0.43, 2.05) | 1.00 (reference) | 0.72 (0.34, 1.53) | 0.30 (0.07, 1.30) |  |
| **Community type** |  |  |  |  |  |  | 0.086 |
| Urban | 159 | 0.29 (0.02, 3.73) | 2.55 (0.73, 8.97) | 1.00 (reference) | 0.81 (0.23, 2.79) | 0.54 (0.08, 3.78) |  |
| Rural | 742 | **0.41 (0.19, 0.88)** | 0.69 (0.42, 1.13) | 1.00 (reference) | **0.54 (0.33, 0.87)** | **0.24 (0.09, 0.67)** |  |
| **Smoking status** |  |  |  |  |  |  | 0.744 |
| Never | 660 | **0.41 (0.17, 0.99)** | 0.98 (0.59, 1.64) | 1.00 (reference) | **0.54 (0.33, 0.91)** | **0.40 (0.16, 1.00)** |  |
| Past and current | 241 | 0.46 (0.13, 1.63) | 0.79 (0.33, 1.87) | 1.00 (reference) | 0.83 (0.35, 1.98) | 0.17 (0.02, 1.63) |  |
| **Drinking status** |  |  |  |  |  |  | 0.325 |
| Never | 615 | 0.64 (0.25, 1.67) | 1.32 (0.76, 2.29) | 1.00 (reference) | 0.70 (0.41, 1.20) | 0.43 (0.17, 1.09) |  |
| Past and current | 286 | 0.35 (0.12, 1.06) | 0.47 (0.22, 1.00) | 1.00 (reference) | 0.48 (0.22, 1.08) | **0.08 (0.01, 0.86)** |  |
| **Nap rhythm** |  |  |  |  |  |  | 0.619 |
| Never | 355 | 0.82 (0.29, 2.28) | 1.02 (0.49, 2.10) | 1.00 (reference) | 0.76 (0.37, 1.53) | 0.26 (0.06, 1.09) |  |
| Had | 546 | **0.23 (0.08, 0.67)** | 0.87 (0.50, 1.54) | 1.00 (reference) | **0.50 (0.29, 0.89)** | 0.36 (0.12, 1.06) |  |
| Body Mass Index |  |  |  |  |  |  | 0.326 |
| <24.00 kg/m^2^ | 527 | 0.50 (0.22, 1.18) | 1.02 (0.58, 1.79) | 1.00 (reference) | **0.47 (0.27, 0.83)** | **0.20 (0.05, 0.72)** |  |
| ≥24.00 kg/m^2^ | 374 | 0.41 (0.11, 1.52) | 0.80 (0.38, 1.68) | 1.00 (reference) | 0.96 (0.46, 2.00) | 0.72 (0.22, 2.38) |  |
| **Physical activity** | |  |  |  |  |  | 0.570 |
| Low | 153 | 0.44(0.00, 79.58) | 0.10 (0.01, 1.01) | 1.00 (reference) | 1.04(0.10, 10.98) | NA |  |
| Middle | 379 | 0.43 (0.14, 1.32) | 0.79 (0.40, 1.56) | 1.00 (reference) | **0.47 (0.23, 0.95)** | 0.35 (0.08, 1.53) |  |
| High | 369 | 0.33 (0.11, 1.01) | 1.46 (0.70, 3.03) | 1.00 (reference) | 0.68 (0.35, 1.31) | 0.32 (0.10, 1.02) |  |
| **Sleep quality** |  |  |  |  |  |  | 0.114 |
| Good | 640 | 0.53 (0.25, 1.14) | 0.97 (0.60, 1.57) | 1.00 (reference) | **0.55 (0.34, 0.89)** | 0.45 (0.19, 1.10) |  |
| Poor | 261 | **0.11 (0.01, 0.99)** | 0.68 (0.22, 2.15) | 1.00 (reference) | 1.28 (0.44, 3.70) | NA |  |
| **Sleep duration** |  |  |  |  |  |  | 0.354 |
| <6h | 184 | **0.03 (0.00, 0.41)** | 0.16 (0.03, 1.02) | 1.00 (reference) | 0.40 (0.08, 2.00) | 0.24 (0.02, 3.07) |  |
| 6h-8h | 545 | 0.62 (0.28, 1.41) | 1.07 (0.64, 1.79) | 1.00 (reference) | 0.89 (0.53, 1.51) | **0.20 (0.04, 0.95)** |  |
| >8h | 172 | **0.05 (0.00, 1.00)** | **0.18 (0.03, 0.99)** | 1.00 (reference) | **0.08 (0.02, 0.43)** | 0.25 (0.04, 1.49) |  |

* All the same covariates as in Model 4 were adjusted.

**Table S5** Stratified analysis for breakfast time and healthy aging

|  |  |  | *OR* (95%*CI*) * |  |  |
| --- | --- | --- | --- | --- | --- |
| Subgroup | N | < 6:00 | 6:00-7:00 | > 6:00 | *P* **_interaction_** |
| **Age** |  |  |  |  | 0.568 |
| ≤70 years | 507 | 0.87 (0.42, 1.81) | 1.00 (reference) | 0.72 (0.46, 1.12) |  |
| >70 years | 394 | 0.49 (0.16, 1.49) | 1.00 (reference) | 0.48 (0.23, 1.00) |  |
| **Sex** |  |  |  |  | 0.365 |
| Male | 391 | 0.58 (0.23, 1.50) | 1.00 (reference) | **0.55 (0.32, 0.96)** |  |
| Female | 510 | 1.04 (0.47, 2.31) | 1.00 (reference) | 0.90 (0.53, 1.50) |  |
| **Education level** |  |  |  |  | 0.8520 |
| Illiteracy | 250 | 0.58 (0.16, 2.06) | 1.00 (reference) | 0.64 (0.27, 1.57) |  |
| Primary and below | 384 | 0.72 (0.28, 1.85) | 1.00 (reference) | **0.49 (0.26, 0.92)** |  |
| Junior high and above | 267 | 0.70 (0.21, 2.41) | 1.00 (reference) | 0.75 (0.39, 1.42) |  |
| **Community type** |  |  |  |  | 0.053 |
| Urban | 159 | 0.13 (0.01, 1.43) | 1.00 (reference) | 0.88 (0.35, 2.26) |  |
| Rural | 742 | 0.93 (0.50, 1.74) | 1.00 (reference) | **0.63 (0.41, 0.96)** |  |
| **Smoking status** |  |  |  |  | 0.061 |
| Never | 660 | 1.18 (0.59, 2.34) | 1.00 (reference) | 0.84 (0.54, 1.30) |  |
| Past and current | 241 | **0.28 (0.08, 0.98)** | 1.00 (reference) | **0.41 (0.19, 0.89)** |  |
| **Drinking status** |  |  |  |  | **0.002** |
| Never | 615 | 1.47 (0.71, 3.05) | 1.00 (reference) | 1.01 (0.63, 1.60) |  |
| Past and current | 286 | **0.30 (0.09, 0.97)** | 1.00 (reference) | **0.27 (0.13, 0.56)** |  |
| **Nap rhythm** |  |  |  |  | 0.753 |
| Never | 355 | 0.65 (0.27, 1.55) | 1.00 (reference) | **0.51 (0.28, 0.94)** |  |
| Had | 546 | 0.87 (0.38, 1.97) | 1.00 (reference) | 0.67 (0.41, 1.10) |  |
| **Body Mass Index** |  |  |  |  | **0.032** |
| <24.00 kg/m^2^ | 527 | 1.07 (0.52, 2.23) | 1.00 (reference) | **0.47 (0.29, 0.78)** |  |
| ≥24.00 kg/m^2^ | 374 | 0.56 (0.19, 1.66) | 1.00 (reference) | 1.03 (0.56, 1.88) |  |
| **Physical activity** | |  |  |  | 0.555 |
| Low | 153 | 0.64 (0.04, 10.71) | 1.00 (reference) | 0.31 (0.05, 1.77) |  |
| Middle | 379 | 1.18 (0.46, 3.02) | 1.00 (reference) | 0.75 (0.41, 1.36) |  |
| High | 369 | 0.69 (0.28, 1.72) | 1.00 (reference) | 0.64 (0.36, 1.13) |  |
| **Sleep quality** |  |  |  |  | 0.286 |
| Good | 640 | 0.82 (0.43, 1.56) | 1.00 (reference) | **0.62 (0.41, 0.94)** |  |
| Poor | 261 | 0.35 (0.07, 1.82) | 1.00 (reference) | 1.08 (0.44, 2.62) |  |
| **Sleep duration** |  |  |  |  | 0.174 |
| <6h | 184 | **0.09 (0.01, 1.00)** | 1.00 (reference) | 1.59 (0.43, 5.81) |  |
| 6h-8h | 545 | 1.01 (0.52, 1.95) | 1.00 (reference) | 0.70 (0.44, 1.12) |  |
| >8h | 172 | NA | 1.00 (reference) | 0.43 (0.14, 1.36) |  |

* All the same covariates as in Model 4 were adjusted.

**Table S6** Stratified analysis for dinner time and healthy aging

|  |  |  | *OR* (95%*CI*)* |  |  |
| --- | --- | --- | --- | --- | --- |
| Subgroup | N | < 17:00 | 17:00-18:00 | > 18:00 | ***P* _interaction_** |
| **Age** |  |  |  |  | 0.769 |
| ≤70 years | 507 | **0.35 (0.12, 0.98)** | 1.00 (reference) | **0.46 (0.26, 0.83)** |  |
| >70 years | 394 | 0.48 (0.16, 1.45) | 1.00 (reference) | 0.33 (0.08, 1.28) |  |
| **Sex** |  |  |  |  | 0.075 |
| Male | 391 | 0.61 (0.21, 1.75) | 1.00 (reference) | 0.92 (0.45, 1.90) |  |
| Female | 510 | 0.35 (0.12, 1.08) | 1.00 (reference) | **0.18 (0.07, 0.45)** |  |
| **Education level** |  |  |  |  | 0.602 |
| Illiteracy | 250 | 0.93 (0.25, 3.49) | 1.00 (reference) | 0.77 (0.21, 2.78) |  |
| Primary and below | 384 | **0.23 (0.06, 0.85)** | 1.00 (reference) | **0.31 (0.13, 0.73)** |  |
| Junior high and above | 267 | 0.68 (0.15, 3.05) | 1.00 (reference) | 0.51 (0.20, 1.26) |  |
| **Community type** |  |  |  |  | 0.417 |
| Urban | 159 | 1.27 (0.29, 5.60) | 1.00 (reference) | 1.15 (0.23, 5.86) |  |
| Rural | 742 | **0.37 (0.15, 0.93)** | 1.00 (reference) | **0.41 (0.23, 0.74)** |  |
| **Smoking status** |  |  |  |  | 0.500 |
| Never | 660 | 0.50 (0.21, 1.19) | 1.00 (reference) | **0.35 (0.18, 0.69)** |  |
| Past and current | 241 | 0.40 (0.09, 1.81) | 1.00 (reference) | 0.57 (0.22, 1.51) |  |
| **Drinking status** |  |  |  |  | 0.860 |
| Never | 615 | 0.54 (0.21, 1.39) | 1.00 (reference) | **0.46 (0.23, 0.93)** |  |
| Past and current | 286 | 0.42 (0.12, 1.50) | 1.00 (reference) | 0.59 (0.25, 1.39) |  |
| **Nap rhythm** |  |  |  |  | 0.789 |
| Never | 355 | 0.73 (0.21, 2.53) | 1.00 (reference) | 0.47 (0.20, 1.11) |  |
| Had | 546 | 0.39 (0.15, 1.00) | 1.00 (reference) | **0.41 (0.21, 0.80)** |  |
| **Body Mass Index** |  |  |  |  | 0.767 |
| <24.00 kg/m^2^ | 527 | 0.50 (0.20, 1.24) | 1.00 (reference) | **0.53 (0.28, 1.00)** |  |
| ≥24.00 kg/m^2^ | 374 | 0.36 (0.09, 1.51) | 1.00 (reference) | **0.33 (0.11, 0.96)** |  |
| **Physical activity** | |  |  |  | 0.277 |
| Low | 153 | NA | 1.00 (reference) | 0.25 (0.02, 3.58) |  |
| Middle | 379 | 0.67 (0.23, 1.99) | 1.00 (reference) | 0.56 (0.24, 1.31) |  |
| High | 369 | 0.43 (0.14, 1.33) | 1.00 (reference) | **0.44 (0.20, 0.98)** |  |
| **Sleep quality** |  |  |  |  | 0.528 |
| Good | 640 | **0.40 (0.17, 0.96)** | 1.00 (reference) | **0.46 (0.25, 0.84)** |  |
| Poor | 261 | 0.99 (0.19, 5.08) | 1.00 (reference) | 0.44 (0.12, 1.65) |  |
| **Sleep duration** |  |  |  |  | 0.467 |
| <6h | 184 | 0.15 (0.01, 2.33) | 1.00 (reference) | 0.42 (0.08, 2.14) |  |
| 6h-8h | 545 | 0.57 (0.21, 1.57) | 1.00 (reference) | **0.33 (0.16, 0.65)** |  |
| >8h | 172 | 0.78 (0.16, 3.79) | 1.00 (reference) | 0.26 (0.04, 1.84) |  |

* All the same covariates as in Model 4 were adjusted.

**Table S7** Odds ratio of healthy aging by nighttime fasting duration, sensitivity analysis

|  |  |  |  | *OR* (95%*CI*) * |  |  |
| --- | --- | --- | --- | --- | --- | --- |
| Limited population | N | NFD<12h | 12h≤NFD<13h | 13h≤NFD<14h | 14h≤NFD<15h | 15h≤NFD |
| Moderately morning and Definitely morning | 823 | **0.41 (0.20, 0.84)** | 0.90 (0.57, 1.41) | 1.00 (reference) | **0.63 (0.40, 0.98)** | **0.29 (0.12, 0.71)** |
| Feeding frequency is three times | 829 | 0.52 (0.25, 1.07) | 1.05 (0.67, 1.64) | 1.00 (reference) | 0.72 (0.46, 1.12) | **0.40 (0.17, 0.92)** |
| No shift work experience | 766 | **0.47 (0.23, 0.96)** | 0.99 (0.62, 1.60) | 1.00 (reference) | 0.66 (0.42, 1.06) | **0.36 (0.14, 0.87)** |
| Moderate and high physical activity level | 748 | **0.44 (0.21, 0.91)** | 1.03 (0.64, 1.64) | 1.00 (reference) | **0.61 (0.39, 0.95)** | **0.38 (0.16, 0.91)** |
| Good sleep quality | 640 | 0.53 (0.25, 1.14) | 0.97 (0.60, 1.57) | 1.00 (reference) | **0.55 (0.34, 0.89)** | 0.45 (0.19, 1.10) |

* All other variables were adjusted for, except the variable used for sensitivity analysis.

**Table S8** Odds ratio of healthy aging by breakfast time, sensitivity analysis

|  |  |  | *OR* (95%*CI*) * |  |
| --- | --- | --- | --- | --- |
| Limited population | N | < 6:00 | 6:00-7:00 | > 7:00 |
| Moderately morning and Definitely morning | 823 | 0.76 (0.42, 1.38) | 1.00 (reference) | **0.62 (0.42, 0.92)** |
| Feeding frequency is three times | 829 | 0.84 (0.46, 1.54) | 1.00 (reference) | 0.69 (0.47, 1.02) |
| No shift work experience | 766 | 0.71 (0.38, 1.32) | 1.00 (reference) | 0.70 (0.47, 1.04) |
| Moderate and high physical activity level | 748 | 0.89 (0.47, 1.66) | 1.00 (reference) | 0.71 (0.48, 1.05) |
| Good sleep quality | 640 | 0.82 (0.43, 1.56) | 1.00 (reference) | **0.62 (0.41, 0.94)** |

* All other variables were adjusted for, except the variable used for sensitivity analysis.

**Table S9** Odds ratio of healthy aging by dinner time, sensitivity analysis

|  |  |  | *OR* (95%*CI*) * |  |
| --- | --- | --- | --- | --- |
| Limited population | N | < 17:00 | 17:00-18:00 | > 18:00 |
| Moderately morning and Definitely morning | 823 | **0.48 (0.23, 1.00)** | 1.00 (reference) | **0.37 (0.21, 0.65)** |
| Feeding frequency is three times | 829 | 0.47 (0.22, 1.03) | 1.00 (reference) | **0.52 (0.30, 0.89)** |
| No shift work experience | 766 | **0.38 (0.16, 0.90)** | 1.00 (reference) | **0.46 (0.27, 0.80)** |
| Moderate and high physical activity level | 748 | 0.56 (0.26, 1.18) | 1.00 (reference) | **0.54 (0.31, 0.94)** |
| Good sleep quality | 640 | **0.40 (0.17, 0.96)** | 1.00 (reference) | **0.46 (0.25, 0.84)** |

* All other variables were adjusted for, except the variable used for sensitivity analysis.


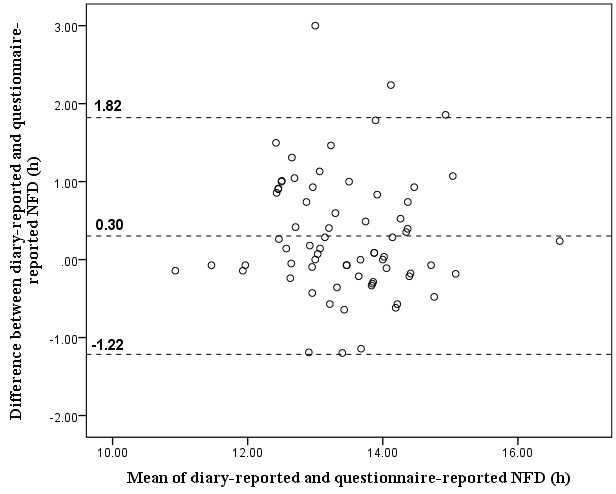


**Fig S1** Bland-Altman plot of diary-reported and questionnaire-reported NFD


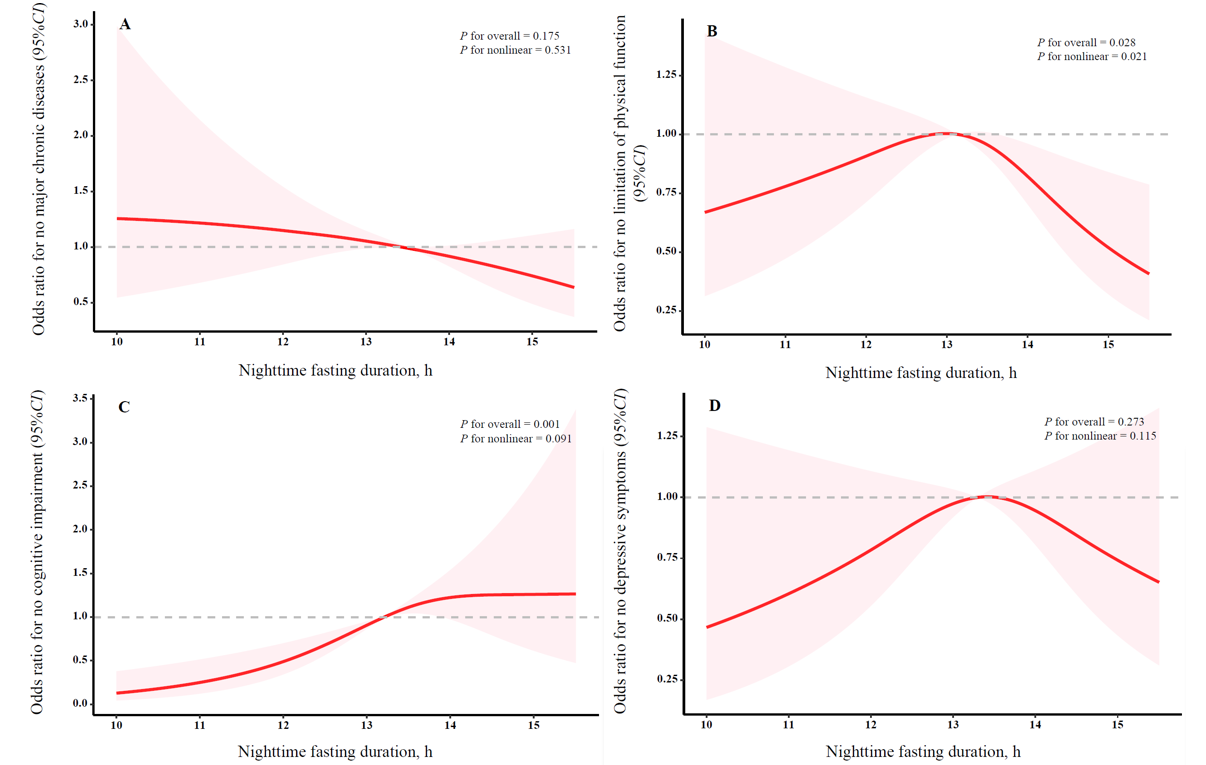


**Fig S2** RCS analyses of NFD in relation to the four dimensions of healthy aging (all the same covariates as in Model 4 were adjusted)


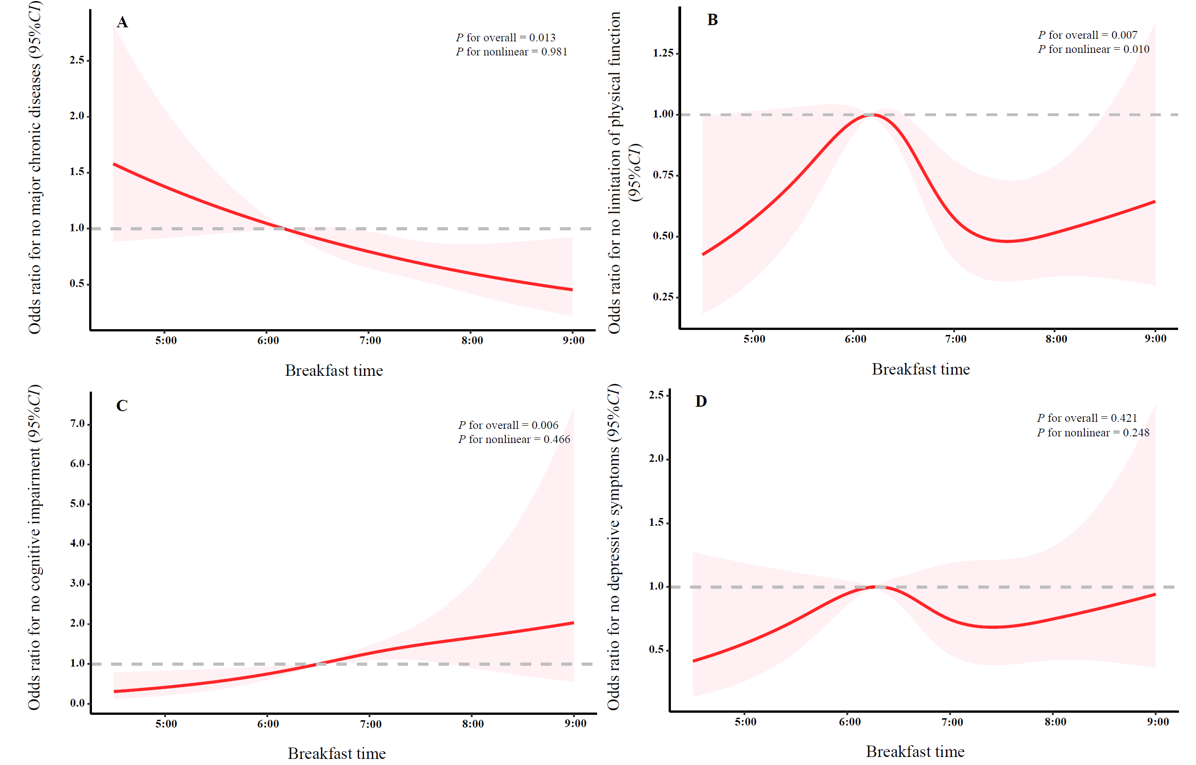


**Fig S3** RCS analyses of breakfast time in relation to the four dimensions of healthy aging (all the same covariates as in Model 4 were adjusted)


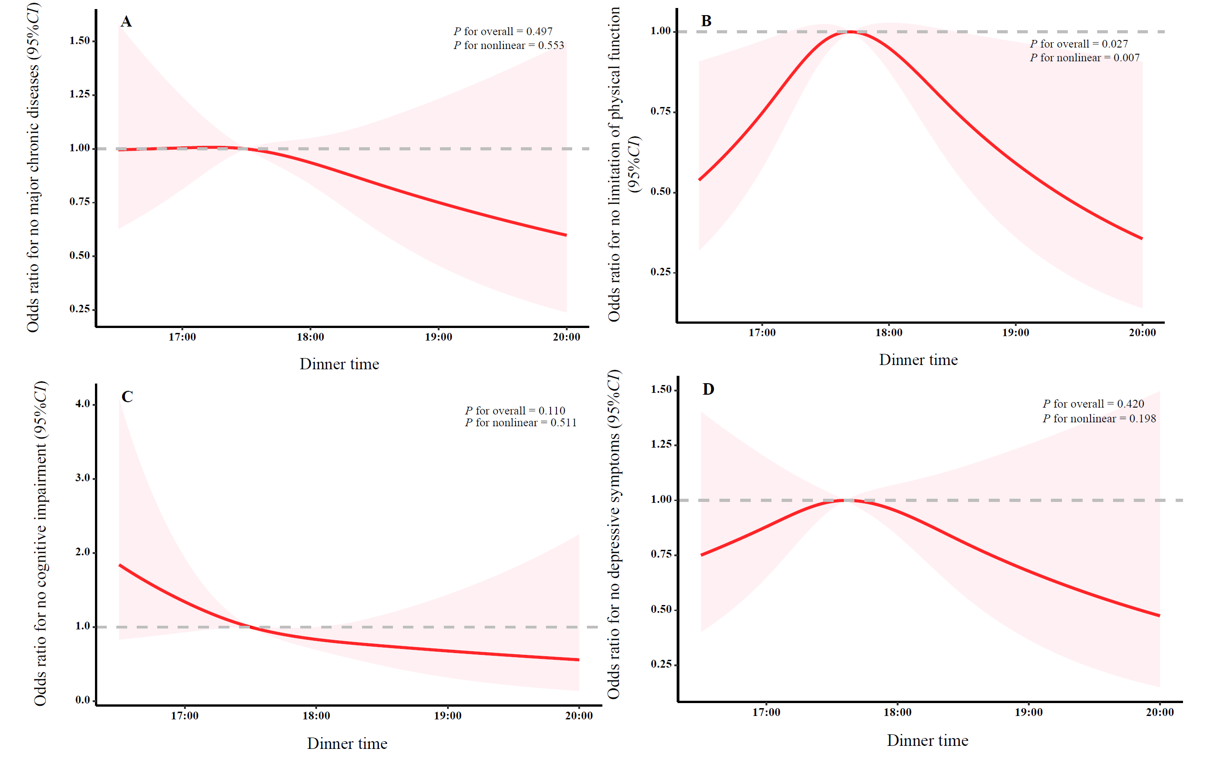


**Fig S4** RCS analyses of dinner time in relation to the four dimensions of healthy aging (all the same covariates as in Model 4 were adjusted)


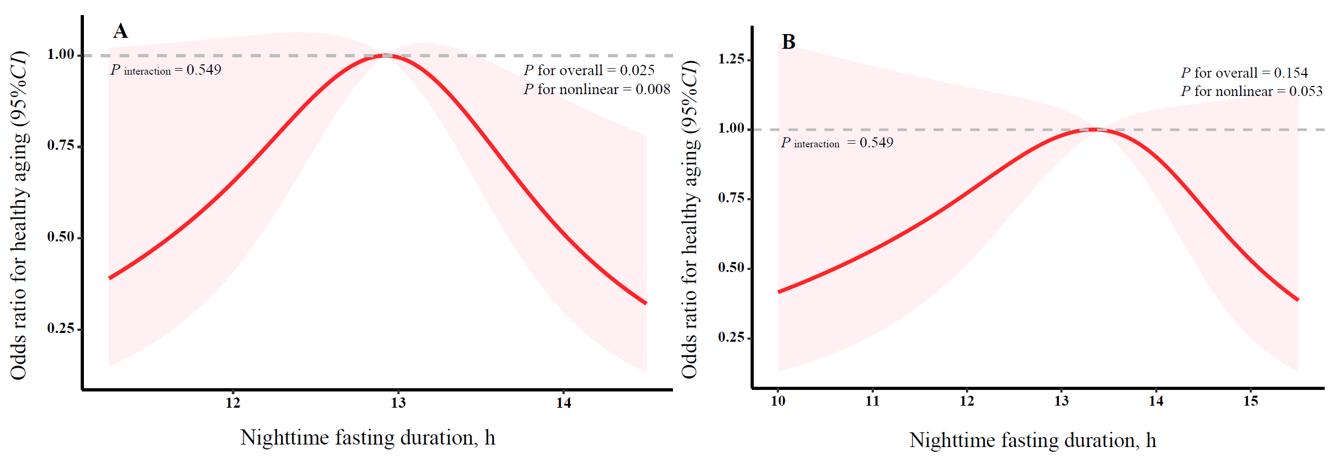


**Fig S5** RCS analyses of NFD and healthy aging according to breakfast time: (A) normal breakfast time (6:00–7:00) and (B) abnormal breakfast time (<6:00 or >7:00), adjusted for the same covariates as in Model 4


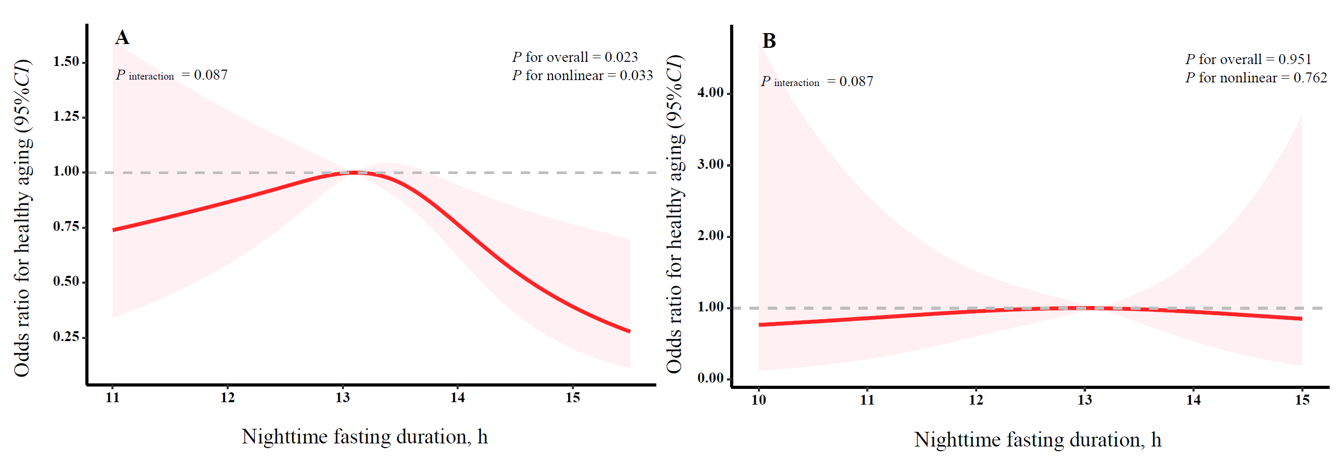


**Fig S6** RCS analyses of NFD and healthy aging according to dinner timing: (A) normal dinner time (6:00–7:00) and (B) abnormal dinner time (<6:00 or >7:00), adjusted for the same covariates as in Model 4


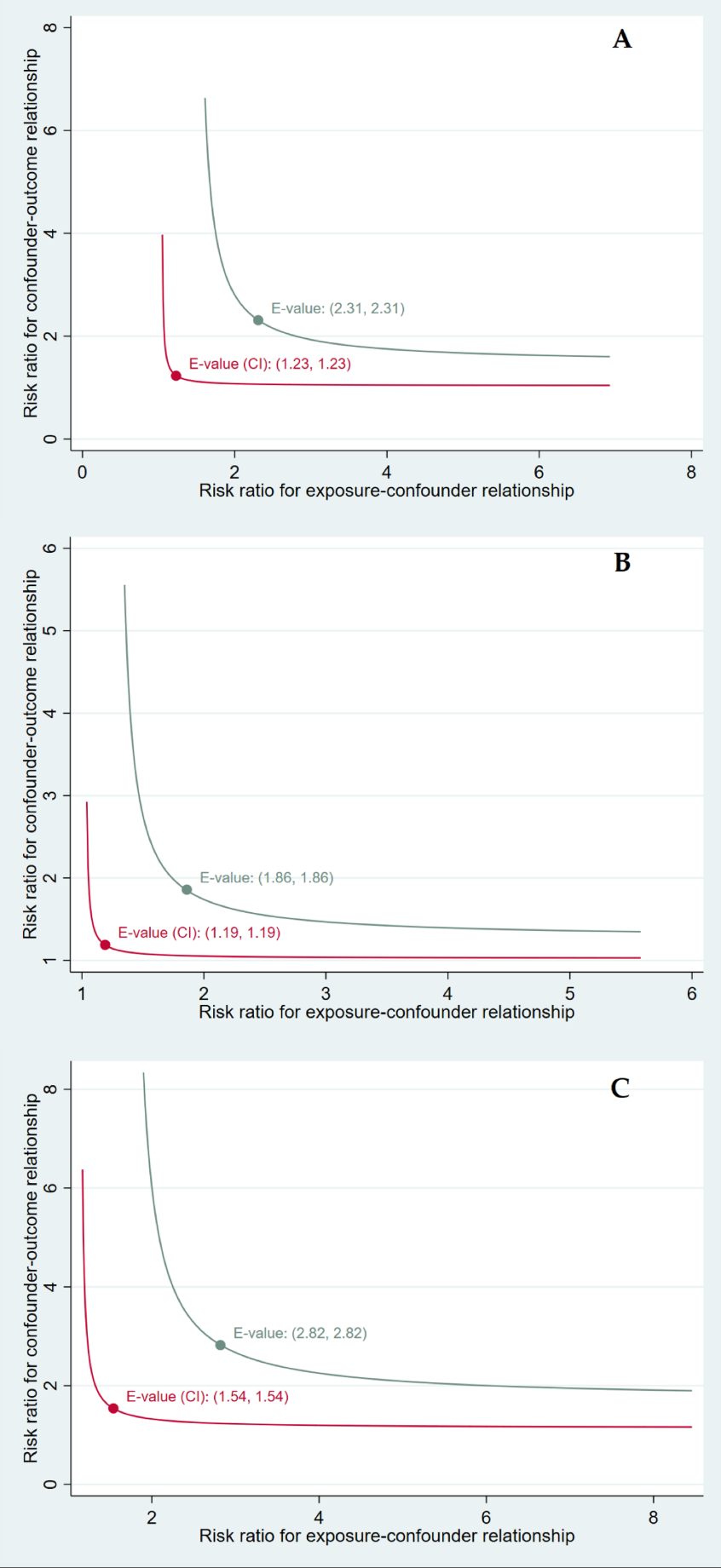


**Fig S7** Curves of the sensitivity analysis for unobserved confounders with E-value highlighted

A: NFD<12h VS 13h≤NFD<14h; B: 14h≤NFD<15h VS 13h≤NFD<14h; C: NFD≥15h VS 13h≤NFD<14h.
